# Patient satisfaction, preferences, expectations, characteristics, and impact of suboptimal control of rheumatoid arthritis: A subgroup analysis of Japanese patients from a large international cohort study (SENSE)

**DOI:** 10.1371/journal.pone.0259389

**Published:** 2021-11-15

**Authors:** Yutaka Kawahito, Yuya Takakubo, Akio Morinobu, Naoko Matsubara, Orsolya Nagy, Eiji Sugiyama

**Affiliations:** 1 Inflammation and Immunology, Graduate School of Medical Science, Kyoto Prefectural University of Medicine, Kyoto, Japan; 2 Department of Orthopaedic Surgery, Yamagata University Faculty of Medicine, Yamagata, Japan; 3 Rheumatology and Clinical Immunology, Kobe University Graduate School of Medicine, Kobe, Japan; 4 Study Design and Data Science Team Evidence Solution Group Medical, AbbVie GK, Tokyo, Japan; 5 Global Medical Affairs, AbbVie, Chicago, Illinois, United States of America; 6 Department of Clinical Immunology and Rheumatology, Hiroshima University Hospital, Hiroshima, Japan; Nippon Medical School, JAPAN

## Abstract

**Objective:**

To evaluate treatment satisfaction, disease outcomes, and perspectives of patients with poorly controlled rheumatoid arthritis (RA) treated with conventional synthetic, targeted synthetic, or biologic disease-modifying antirheumatic drugs (DMARDs), we conducted a subgroup (post hoc) analysis of Japanese patients participating in the SENSE study.

**Methods:**

Data for Japanese patients (n/N = 118/1629) from the global, multicenter, cross-sectional, observational SENSE study were analyzed. The primary endpoint was the global satisfaction subscore assessed using the Treatment Satisfaction Questionnaire for Medication (TSQM) version 1.4. Other patient-reported outcomes included self-reported RA medication adherence and Work Productivity and Activity Impairment-RA. Patient perspectives included patients’ expectations and preference of pharmacologic treatment.

**Results:**

Median (range) age and RA disease duration were 67.0 (18.0–87.0) years and 8 (0.0-54) years, respectively; 81.4% of patients were female. Mean (SD) TSQM global satisfaction subscore was 56.8 (17.5), and only 5.9% of patients reported good satisfaction with treatment (TSQM global ≥80). Mean (SD) self-reported treatment adherence using VAS was high (93.5% [13.8%]). Mean (SD) total work productivity impairment was 45.6% (32.0%); presenteeism contributed toward more total work productivity impairment (43.9% [30.4%]) than absenteeism (8.3% [24.4%]). Patients expected improvement in all parameters from their treatment, especially improvement in joint symptoms. Most patients (80.7%) preferred oral medication and 18.7% preferred monotherapy. Patient acceptability of potentially manageable side effects was high (7.5%-34.0%). Although most patients (81.3%) found combination therapy acceptable, 43.2% were receiving DMARD monotherapy.

**Conclusion:**

Although most Japanese patients with RA with moderate-to-high disease activity were dissatisfied with their current DMARD treatment, high treatment adherence, high acceptability of combination therapy, high acceptability of manageable potential side effects, and preference for oral medication were reported. Data support the development of a more individualized and patient-centric approach for RA treatment.

## Introduction

Rheumatoid arthritis (RA) is a global public health challenge with increasing prevalence and incidence rates worldwide [1] as well as a negative impact on patients’ quality of life and ability to work [2]. The estimated prevalence of RA in Japan ranges from 0.6% to 1.0% according to the Institute of Rheumatology, Rheumatoid Arthritis (IORRA) registry [3]; the peak age at onset during 2012–2013 was between 60 and 69 years according to the National Database of Rheumatic Diseases by iR-net in Japan [4].

The evolution of the disease-modifying antirheumatic drugs (DMARDs) has provided wider treatment options for RA, including conventional synthetic DMARDs (csDMARDs), biologic DMARDs (bDMARDs), and targeted synthetic DMARDs (tsDMARDs) [5]. Moreover, recommendations on the treat-to-target (T2T) approach for achieving optimal therapeutic outcomes in RA aimed at remission or low disease activity were published by the European League Against Rheumatism (EULAR) in 2010 [6] and updated in 2014 [7].

However, important differences in RA management have been noted in Japan compared with other countries. Methotrexate (MTX) was approved in Japan more than a decade after it had been approved in western countries [8], and is traditionally prescribed at lower doses in Japan compared with other regions [9]. Hydroxychloroquine has not been approved for the treatment of RA in Japan [9]. The immunosuppressant tacrolimus and csDMARDs (such as bucillamine and iguratimod) were developed in Japan. Among these, the use of tacrolimus is largely limited to Asia and Canada [9].

Compared with csDMARDs, Japanese physicians can prescribe nearly all bDMARDs for the treatment of RA, with the exception of the interleukin-1 antagonist anakinra and the B-cell–targeted anti-CD20 monoclonal antibody rituximab [9]. The national health insurance system in Japan ensures that advanced therapies, such as bDMARDs and tsDMARDs, are more easily available to patients with RA compared with that in other countries [9]. Furthermore, in Japan, rheumatologists and orthopedic surgeons treating RA are reluctant to use glucocorticoids for the long-term treatment of RA owing to their adverse effects, such as an increased risk of infection or osteoporosis [9].

Despite the availability of a wide range of treatment options, many patients with RA remain suboptimally managed, and patients’ needs are frequently unmet across outcomes such as improvement in pain and fatigue. This can adversely affect patients’ adherence, social functioning, and ability to work [10–16]. Patient satisfaction, as an essential outcome, is closely linked to treatment adherence and persistence [15] but is not well studied in patients with RA with suboptimal disease control.

Research shows that in Japanese patients with RA, disease activity does not correlate with psychosocial factors and is disconnected from the perceived physical or mental quality of life of the patients [16]. Therefore, assessing patients’ perspectives enhances understanding of the unmet needs and treatment benefits [17, 18]. Moreover, the EULAR 2014 T2T recommendations state that shared decision-making between the patient and rheumatologist is essential and that the patient should be involved in setting the treatment target and strategy [7]. Research is therefore warranted to understand patients’ expectations and preferences of the current RA pharmacologic approaches to enable successful treatment. Although digital health literacy influences patient adherence, empowerment, and self-care [19], it has not been assessed in large groups of patients with RA using a validated tool.

Consequently, the SENSE (Cross-sectional **S**tudy **E**valuating patie**N**t satisfaction, adherence feature**S**, and th**E**ir association with sociodemographic and clinical characteristics of DMARD-inadequate responder rheumatoid arthritis patients) study was designed to assess treatment satisfaction, disease outcomes, and perspectives of patients with poorly controlled RA treated with csDMARDs, tsDMARDs, or bDMARDs [20, 21]. Here, we present the results of the subgroup (post hoc) analysis of Japanese patients who participated in the SENSE study.

## Patients and methods

### Study design and patients

Data from Japanese patients (n = 118) enrolled in the multicenter, cross-sectional, observational SENSE study (N = 1629) conducted in community- or hospital-based medical centers globally (18 countries) were analyzed. The study design and overall results have been previously reported [21]. Patients were enrolled from September 14, 2018, to May 31, 2019. Briefly, the study population comprised adults (aged ≥18 years) with poorly controlled RA, defined as a Disease Activity Score in 28 joints (DAS28) of >3.2 for 1–4 months, despite receiving a full tolerable dosage of current DMARD for ≥3 months. Other key inclusion criteria were RA, diagnosed using either the 1987 revised American College of Rheumatology (ACR) or the 2010 ACR/EULAR classification criteria for RA [22, 23]; current treatment with any approved csDMARD, tsDMARD, or bDMARD; exposure to ≤2 bDMARDs at the time of enrollment; willingness/ability to complete patient-reported outcome (PRO) questionnaires; no participation in any other clinical study for RA; and willingness to provide written authorization to the investigator to use and/or disclose personal and/or health data if requested by local regulations. The study was performed in accordance with the Guidelines for Good Pharmacoepidemiology Practices and approved by the institutional review board at each study site. All patients provided written informed consent before enrollment.

### Outcome measures and assessments

Patient demographics and characteristics, including details of occupation and disease activity, were recorded. Further details have been presented previously [21].

#### PRO measures

The primary outcome measure of this study was patient satisfaction with RA treatment, assessed using the 14-item Treatment Satisfaction Questionnaire for Medication (TSQM) version 1.4, which evaluated four domains (effectiveness, side effects, convenience, and global satisfaction) [24]. Good treatment satisfaction was defined as a global satisfaction score ≥80 out of a possible 100 [17].

#### Other PRO/secondary outcome measures

Other PRO/secondary outcome measures included physical function assessed using the 20-item Health Assessment Questionnaire-Disability Index (HAQ-DI), with scores ranging from 0 to 3 (higher values indicate a greater degree of disability); 13-item Functional Assessment of Chronic Illness Therapy-Fatigue (FACIT-F) scale (4-point Likert scale; a higher FACIT-F score indicates a greater quality of life) [25]; severity of morning stiffness (0–10, visual analog scale [VAS]) and duration of morning stiffness (hours); worst joint pain (0–10, NRS); self-reported RA medication adherence (0–100 mm, VAS; good adherence, ≥80% on the VAS) [26]; Work Productivity and Activity Impairment-RA based on four outcomes [27]; health-related quality of life measured using the 36-item Short-Form Health Survey (SF-36) version 2, Physical Component Summary, and Mental Component Summary (MCS) [28]; and digital health literacy, via the eHealth Literacy Scale (eHEALS, a measure of consumers’ knowledge, comfort, and perceived skills at finding, evaluating, and applying electronic health information to health problems) [29, 30]. Further details have been presented previously [21].

### Patient perspectives

Patient perspectives included patients’ expectations from pharmacologic treatment (11-item questionnaire with a 7-point scale [ranging from 1 = no improvement needed to 7 = the most improvement needed]); patients’ preference of pharmacologic treatment (a questionnaire with six discrete items evaluating combination therapy, route of administration, time to onset of treatment effect, and acceptable side effects); and need for patient support programs (PSPs; 17-item questionnaire with a 7-point scale [ranging from 1 = not needed at all to 7 = very much needed]). All patient perspective questionnaires were developed by AbbVie owing to the limited availability of appropriate validated tools [21].

### Procedures

Treatment was based on the treating physician’s discretion. Data collection was performed using an electronic case report form (eCRF) during a single prescheduled visit for a routine clinical follow-up. Each site documented patient data in the eCRF in the electronic data capture (EDC) system. Data were entered by the researcher or staff into the eCRFs according to the study protocol. The information from paper-based patient questionnaires was also entered into the EDC system by site staff. The eCRFs for investigators were developed in English; this included PROs and other questionnaires. All questionnaires were provided to patients in their local language/Japanese. This study was conducted in compliance with the ethical principles of the “Declaration of Helsinki” and “Ethical Guidelines for Medical and Health Research Involving Human Subjects.”

### Statistical analyses

The full analysis set was defined as all enrolled patients who fulfilled all the selection criteria. A descriptive analysis was performed, and quantitative data are described as valid N, missing N, mean, standard deviation (SD), median, minimum, maximum, lower quartile (25%), and upper quartile (75%). Qualitative data are presented as means of (absolute and relative) frequency distributions. Two-sided 95% confidence intervals were calculated, as appropriate. P-values less than 0.05 were regarded as statistically significant. All statistical analyses were performed using SAS^®^ software version 9.4. A multiple logistic regression analysis was conducted to determine variables that might predict good treatment satisfaction. For regression analysis, missing/unknown data for prognostic factors were imputed using a regression-based single imputation method, in which missing values of one predictor variable were predicted using regression models, with a set of remaining potential prognostic variables as independent variables. Regression analyses were evaluated for the Japanese subpopulation (which included all eligible patients fulfilling the inclusion criteria) and were conducted on imputed data. The potential prognostic factors used for regression models were specified, and for simplified interpretation, categorical predictors were dichotomized/grouped. For stepwise automatic selection of relevant predictors, backward elimination with a significance level of 0.05 was applied. Dependent variables used in separate multivariate logistic regression models are presented in **S1 Table in** S1 File. Further details are presented in a previous publication [21].

## Results

### Demographics and patient characteristics

Overall, 118 of the 1629 patients in the SENSE study were enrolled from nine study sites in Japan. The full analysis set comprised 118 patients from the Japanese subpopulation. The median (range) age was 67.0 (18.0–87.0) years, and most patients (81.4%) were female (Table 1). Owing to their RA, 13.6% of patients had taken early retirement, compared with 22.0% of those who had a regular retirement, and 4.2% were unemployed. The median (range) RA disease duration was 8.0 (0.0–54.0) years, and approximately half of the patients were full-time employees. The mean (SD) DAS28-C-reactive protein, DAS28-erythrocyte sedimentation rate, Simple Disease Activity Index (SDAI), and Clinical Disease Activity Index (CDAI) scores mostly represented moderate-to-high disease activity in the Japanese subpopulation (Table 1). Given that the data were available for 106–112 of the 118 Japanese patients, the dropout rate was 5.1%-10.2%.

**Table 1:** Patient clinical and sociodemographic characteristics (Japanese subpopulation).

Characteristics	Japanese patients (n = 118)
Age (years), median (range)	67.0 (18.0–87.0)
Duration of RA (years), median (range)	8.0 (0.0–54.0)
Sex (female), n (%)	96 (81.4)
Employed (full-time), n (%)	51 (43.2)
TJC28, mean (SD)	5.1±4.8
SJC28, mean (SD)	5.1±4.7
Patient global assessment (0–10 cm on VAS), mean (SD)	4.6±2.0
Physician global assessment (0–10 cm on VAS), mean (SD)	4.1±2.1
DAS28-CRP, mean (SD)	4.0±0.9
DAS28-ESR (n = 91), mean (SD)	4.6±1.1
CDAI, mean (SD)	18.9±10.0
SDAI, mean (SD)	20.0±10.9
Worst joint pain (0–10, NRS; n = 117), mean (SD)	4.9±2.7
Severity of morning stiffness (0–10, NRS; n = 117), mean (SD)	3.7±2.8
HAQ-DI score (0–3), mean (SD)	1.3±0.8
WPAI-RA subscores (%)	
Presenteeism (n = 44), mean (SD)	43.9±30.4
Absenteeism (n = 42), mean (SD)	8.3±24.4
Total work productivity impairment (n = 41), mean (SD)	45.6±32.0
Total activity impairment (n = 113), mean (SD)	48.0±29.4
eHEALS (8–40; n = 107), mean (SD)	23.4±5.7
Self-reported treatment adherence (0%-100% on VAS; n = 117), mean (SD)	93.5±13.8
SF-36 PCS (0–100; n = 117), mean (SD)	39.3±7.8
SF-36 MCS (0–100; n = 117), mean (SD)	45.5±9.7

Data are presented as mean±SD unless otherwise specified.

CDAI, Clinical Disease Activity Index; CRP, C-reactive protein; DAS28, Disease Activity Score in 28 joints; eHEALS, eHealth Literacy Scale; ESR, erythrocyte sedimentation rate; HAQ-DI, Health Assessment Questionnaire-Disability Index; MCS, Mental Component Summary; NRS, numeric rating scale; PCS, Physical Component Summary; RA, rheumatoid arthritis; SD, standard deviation; SDAI, Simplified Disease Activity Index; SF-36, 36-Item Short-Form Health Survey; SJC28, Swollen Joint Count of 28 joints; TJC28, Tender Joint Count of 28 joints; VAS, visual analog scale; WPAI, Work Productivity and Activity Impairment.

### Medication

Of the 118 patients, most received DMARD therapy (**S2 Table in** S1 File): 43.2% monotherapy (without combining with another DMARD) and 44.9% as a combination of two DMARDs concurrently (one patient was currently not receiving any DMARD). The most commonly used DMARD was MTX (62.7%), followed by sulfasalazine (21.2%) and abatacept (12.7%) (**S3 Table in** S1 File). Only approximately one-third of the patients received advanced therapy (bDMARD, 35.6%; tsDMARD, 5.1%). Most patients receiving a bDMARD (n = 42) or tsDMARD (n = 6) received MTX or another csDMARD(s) as a concomitant medication, except eight and one patient, respectively, who received them as monotherapy (**S2 Table in** S1 File). In the monotherapy group, 82.4% (42/51) of patients received csDMARDs, implying that patients on monotherapy were treated less aggressively compared with those on multiple DMARD combination therapy. Systemic glucocorticoids were used in a small proportion of patients (20.3%; 24/118). A treatment switch to a different DMARD was planned in 38.1% of the total 118 patients.

### PROs

**Fig 1:**
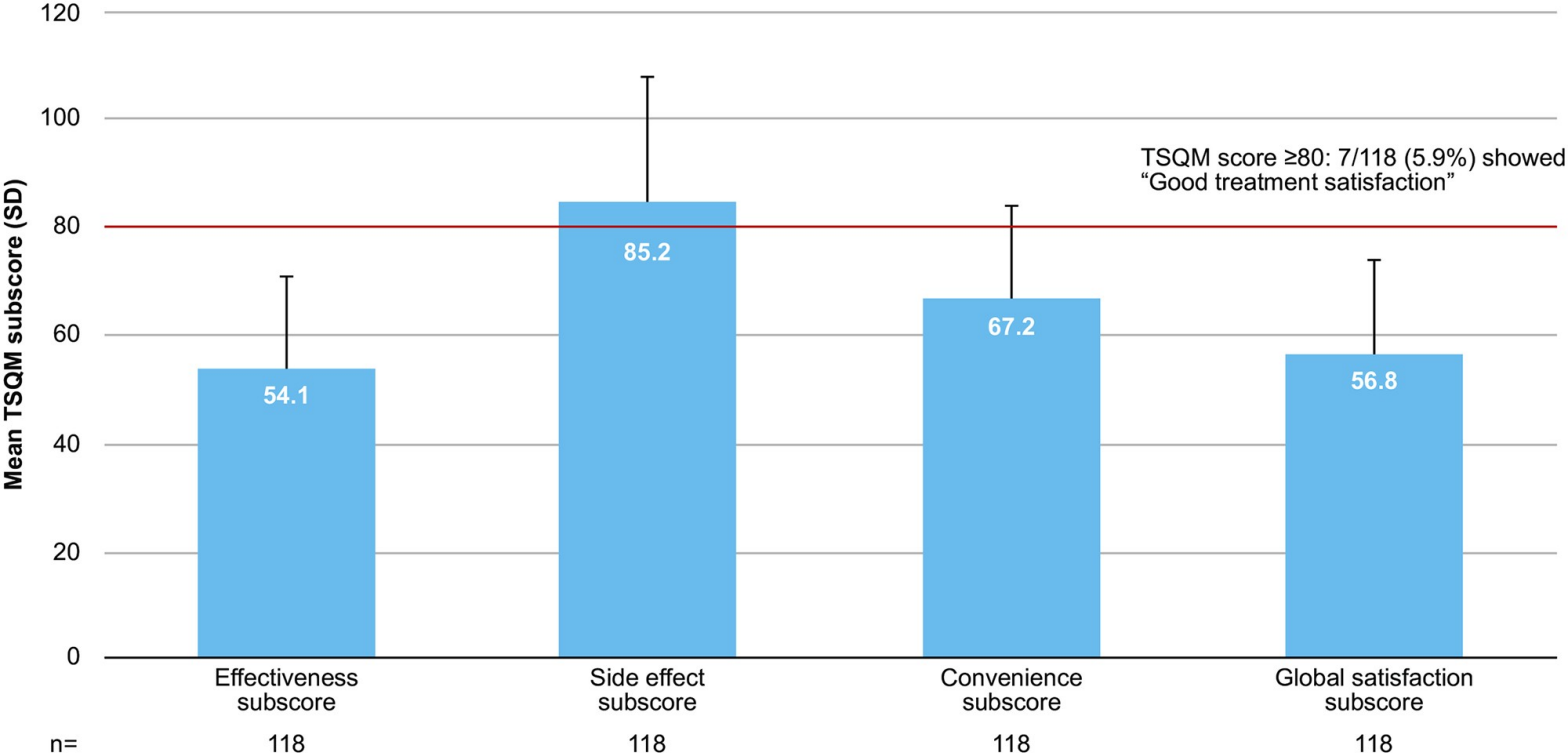
RA treatment satisfaction (Japanese subpopulation). RA, rheumatoid arthritis; SD, standard deviation; TSQM, Treatment Satisfaction Questionnaire for Medication.

Mean (SD) TSQM global satisfaction subscore was 56.8 (17.5), and only 5.9% of the patients reported good satisfaction with treatment (TSQM global ≥80) [17], mainly driven by a low effectiveness subscore (54.1) compared with the dissatisfaction likely due to a high side effect subscore (85.2) (Fig 1). Predictors for good satisfaction were higher number of comorbidities (odds ratio [OR] [95% CI], 2.25 [1.17, 4.34]), higher SF-36 MCS scores (OR, 1.27 [1.08, 1.49]), and male gender (OR for female gender, 0.08 [0.008, 0.86]; p<0.05 using Chi-square test).

Mean (SD) self-reported treatment adherence using VAS was high (93.5% [13.8%]), and good treatment adherence (≥80%) was reported in 89.7% of 117 patients who responded (**S4 Table in** S1 File). Among those who were employed, the median (IQR) and mean (SD) total work productivity impairment was 40.0% (20.0–70.0) and 45.6% (32.0%). Presenteeism contributed toward more total work productivity impairment (mean [SD], 43.9% [30.4%]) than absenteeism (mean [SD], 8.3% [24.4%]) (Table 1). The ability to use online health information to solve health problems was poor (defined as an eHEALS score of <26) [31] in most patients (70.1%) (**S4 Table in** S1 File).

### Patient perspectives

**Fig 2:**
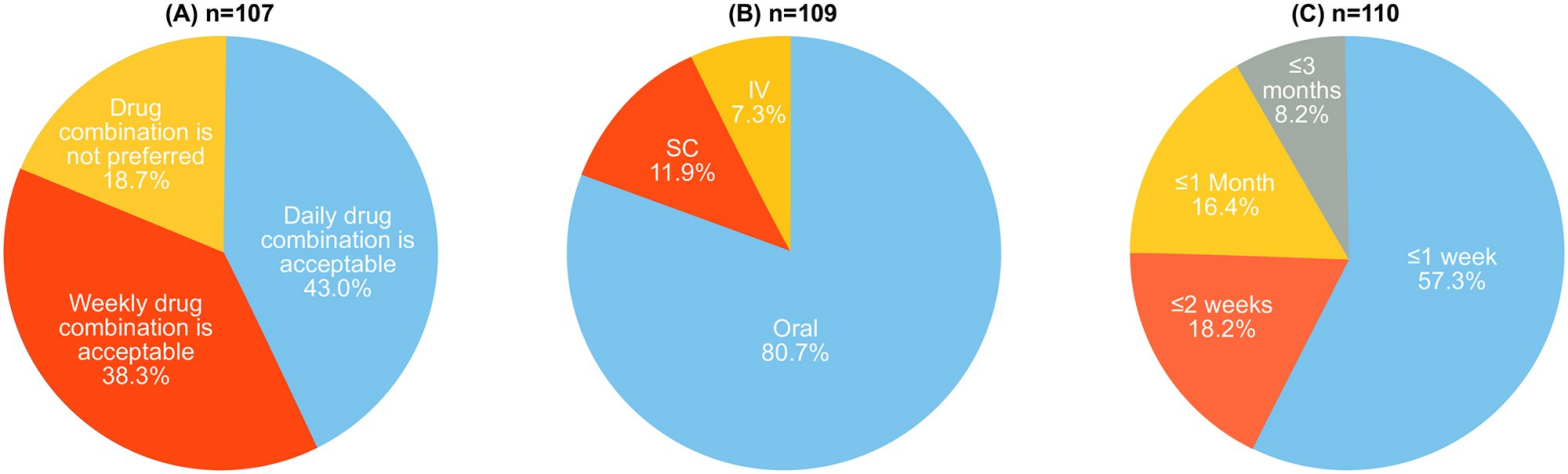
Preferences. (A) Combination therapy, (B) route of administration, and (C) time to effect (Japanese subpopulation). IV, intravenous; SC, subcutaneous.

Patients expected an improvement in all 11 parameters from their treatment with the highest expectation of “less joint pain,” followed by “general improvement of arthritis,” “less joint swelling,” “lasting relief of RA symptoms,” and “more joint flexibility” (Table 2). Most patients (80.7%) preferred oral medication, and more than half (57.3%) of the patients expected the onset of treatment effect within 1 week. A total of 81.3% of patients were willing to accept combination therapy for the treatment of RA (Fig 2).

**Table 2:** Expectations from pharmacologic treatment (Japanese subpopulation).

Expectations	Number of patients who responded	Mean score	SD
General improvement of arthritis	111	6.2	1.4
Less joint pain	110	6.3	1.3
Less joint swelling	111	6.1	1.4
Lasting relief of RA symptoms	111	6.0	1.5
More joint flexibility	110	5.9	1.6
Improvement in morning stiffness in the limbs	110	5.2	2.0
Less tiredness and less fatigue	110	5.1	2.0
Improvement in workability	110	4.7	1.9
Improvement in mood	111	4.6	2.2
Improvements in sleep	111	4.5	2.2
Improvement in self-care	109	4.4	2.1

RA, rheumatoid arthritis; SD, standard deviation.

**Fig 3:**
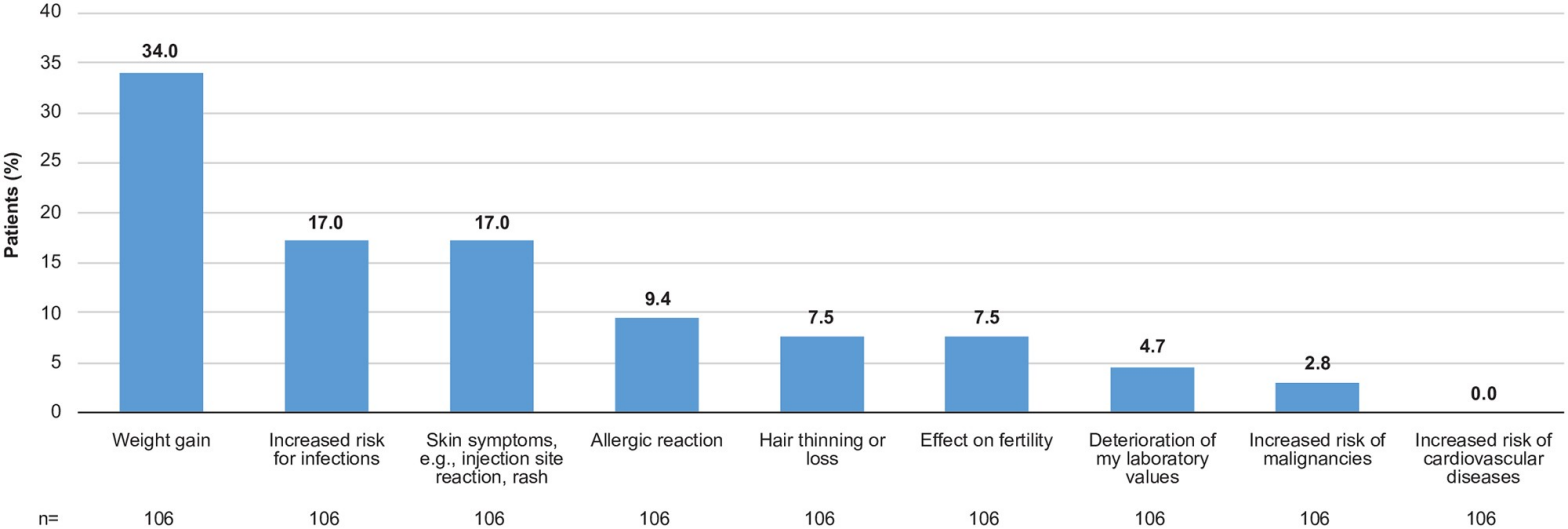
Acceptability of potential side effects of rheumatoid arthritis medication (Japanese subpopulation).

Patient acceptability of potentially manageable side effects ranged widely, with 7.5%-34% of 106 patients reporting it as “acceptable,” depending on the type of side effect; weight gain (34%), followed by skin symptoms (17%), increased risk of infections (17%), allergic reactions (9.4%), hair thinning or loss (7.5%), and effect on fertility (7.5%) had a relatively high acceptability. In contrast, an increased risk of cardiovascular diseases, malignancies, and abnormal laboratory evaluations were side effects acceptable by none or only a minority of the patients—0.0%, 2.8%, 4.7%, respectively, of 106 patients (Fig 3). Most patients (94.6% [106/112]) responded that they needed some kind of support (mean score ≥2), and 77.7% (87/112) had a mean score ≥5 on a scale of 1–7, indicating a strong need for support. In terms of types of patient support needed, responders assigned the greatest importance to educational material on RA, educational material related to treatment for RA, and coaching for personalized care, followed by need for nursing services (Table 3).

**Table 3:** Need for a patient support program (17-item questionnaire with a 7-point scale [ranging from 1 = not needed at all to 7 = very much needed]; Japanese subpopulation).

Variable	Number of patients who responded	Mean score
Need of educational materials about RA disease	112	4.2
Need of educational materials about the therapy the patient takes for RA	112	4.2
Need of a personalized care coach	110	4.2
Need of nursing service	112	4.1
Need of information about everyday coping with the disease	111	4
Need of a starter pack with all information about the patient support program	110	3.9
Need of a patient support program in general	112	3.8
Need of a call center/hotline	112	3.7
Need of educational materials about everyday coping with the disease	111	3.7
Need of doctor appointment reminders	111	3.6
Need of an e-mail contact during the patient support program	110	3.5
Need of medication administration reminders	111	3.4
Need of mental or emotional support	111	3.3
Need of a website where all information related to the patient support program is available	111	3.3
Need of a smartphone application related to the patient support program	111	3.3
Need of a social media communication channel related to the patient support program	111	3.2
Need of digital lifestyle intervention	110	3.1

RA, rheumatoid arthritis.

## Discussion

Understanding of patients’ perspectives and unmet needs of current treatments is essential to enable shared decision-making and adopt a T2T approach in RA. Suboptimal disease control in RA has a significant negative impact on patients’ treatment satisfaction, working life, PROs, and patient perceptions. Results of this subgroup analysis of 118 Japanese patients who participated in the international, multicenter SENSE study [21] showed that this Japanese subpopulation with moderate-to-high disease activity has dissatisfaction of current treatment due to treatment effectiveness, but has high self-perceived treatment adherence, the highest level of acceptance relating to manageable side effects. We believe these results demonstrate the importance of treatment modifications and their education to improve patients’ disease, leading to satisfaction.

The dropout rate in this study was 5.1%-10.2%, which was higher than that in the global study (0.5%) [32], indicating missing data in the Japanese population. The Japanese subpopulation had a higher mean (SD) age (62.8 (15.9) vs 58.4 (13.1) years), more full-time employees (43.2% vs 26.2%), and fewer patients taking regular retirement (22.0% vs 33.8%) compared with the overall global study population in the SENSE study [21]. The impact of an inadequate response to DMARDs on treatment satisfaction and patients’ perspectives on disease management was unique to the Japanese subpopulation compared with the overall global study population [21]. First, a lower proportion of the Japanese subpopulation reported good treatment satisfaction (5.9% vs 13.5%) despite lower disease activity compared with the overall global study population. Predictors for good satisfaction were higher number of comorbidities, higher SF-36 MCS scores, and male gender. As patients with many complications understand that treatment is difficult, we believe that they may report higher satisfaction with the effectiveness of the treatment, given their situation. Second, a greater proportion of the Japanese subpopulation showed acceptance for combination therapy (81.3% vs 18.7% of patients preferred not to use drug combination) compared with the overall global study population (68.6% and 31.3%, respectively). However, nearly half of the patients were on monotherapy. In the monotherapy group, 82.4% (42/51) of patients were treated with a single csDMARD, indicating that the monotherapy group was generally less aggressively treated with any advanced medication. These results suggest that patients on DMARD monotherapy in Japan can receive improved treatment by switching to another DMARD monotherapy or DMARD combination therapy; however, economic background and limitations that prevent the use of expensive therapeutic targeted agents due to comorbidities should be considered. Results from the SENSE global study indicated that a treatment switch was planned in a significantly lower proportion of patients in Asia (including Japan) than in South America (p = 0.01) or Europe (p = 0.01) [21]. Overall, 80.7% of the Japanese subpopulation preferred an oral route of administration compared with 60.7% of the overall global study population. A rapid treatment effect (up to 1 week) was expected by 57.3% of the Japanese subpopulation compared with 71.1% of the overall global study population. Of note, the median disease duration (range) was 8 (0.0–54) years and the mean self-reported treatment adherence was high (93.5%) in the Japanese subpopulation, suggesting that patients were compliant with their prescribed treatment despite poor disease control and a low level of satisfaction. Therefore, shared decision-making when changing treatment, with consideration of patients’ expectations, may lead to improved treatment among Japanese patients with RA.

The acceptability of potentially manageable side effects was high in the Japanese subpopulation. Interestingly, an increased risk for infections was among the top three acceptable side effects in the Japanese subpopulation (17.0%), which was considered less acceptable in the overall study population (9.1%, ranked sixth). This could be attributed to the fact that Japanese patients might be willing to accept side effects that can be controlled by a physician, suggesting that patients do rely on their physician for advice regarding their illness. While the patients’ acceptability was high for manageable side effects, acceptability was low for potentially life-threatening side effects, such as laboratory value deterioration, malignancies, and cardiovascular diseases. Concerns about the risk of side effects are also among the main reasons for unwillingness among Japanese patients with RA to switch their current treatment [33]. Thus, physicians are advised to consider the concerns about side effects and use informative shared decision-making tools about risks and benefits when discussing treatment options with their patients.

Among those who were employed, the median total work productivity impairment (%) was lower in the Japanese subpopulation (40.0) than in the overall global study population (50.0) [21] despite the moderate-to-high disease activity, which was relatively lower in the Japanese subpopulation compared with the overall study population. This could be explained by the Japanese work environment, as presenteeism (mean [SD], 43.9% [30.4%]) contributed toward more total work productivity impairment than absenteeism (mean [SD], 8.3% [24.4%]). It is also known that work disability/work productivity is associated with disease activity and the duration of RA [2, 27, 34, 35]. Biologics have been shown to reduce absenteeism and improve presenteeism [36]. Therefore, more advanced therapy is likely to contribute to increased work productivity.

Most patients (94.6%) responded that they needed some kind of support (mean score ≥2), and 77.7% had a mean score ≥5 on a scale of 1–7, indicating a strong need for support; the greatest importance was assigned by patients to educational material on RA disease, those offering information on the treatment for RA, and personalized care coaching, followed by nursing services. This suggests that patients are positively motivated to seek knowledge regarding RA. The high mean scores for the need for personalized care coaching (4.2) and nursing services (4.1) in the Japanese subpopulation indicated that Japanese patients prefer support from RA specialists. However, patients also reported a poor ability to use available online health information to help solve health problems, as indicated by the eHEALS score of <26 in most patients (70.1%). This further reinforces the finding that patients would benefit from a more proactive approach by a healthcare professional rather than through online guidance. Alternatively, digital tools could be tailored per the needs and capabilities of the RA population.

Patients’ highest treatment expectations were related to joint symptoms. These results were consistent with the findings of a Japanese RA patient survey on treatment expectations, in which most participants placed suppression of or recovery from joint destruction as one of the most important treatment goals [37].

Overall, the results for the Japanese subpopulation indicated that the perception and unmet needs of RA treatment in patients with moderate-to-high disease activity may differ by region or country. These results were largely consistent with previous findings of a pilot, multicenter study of patients with RA, in which treatment expectations varied across China, Japan, and the United States [38]. However, it should also be noted that the ease of choice of RA treatment, other than MTX, was different between the Japanese subpopulation and the overall global study population. Our findings highlight the need for a tailored approach depending on region- or country-specific situations, such as available treatment options, patient-physician relationship, and access to PSPs. Further, according to a cross-sectional, self-administered survey of physicians and surgeons involved in RA treatment in Japan, patient satisfaction was ranked as the most reliable method for assessing the quality of treatment [39]. Importantly, our results indicated an unmet need to improve treatment satisfaction in patients with moderate-to-high disease activity.

### Limitations

The limitations of this study include the fact that the results may not be generalizable to the general Japanese RA population, as this study included only patients with poorly controlled disease and a willingness to participate in clinical research. Additionally, a selection bias, (recall and apprehension) may exist because of the nature of the sampling method and self-reported assessments. Further, the questionnaires used for PSPs, treatment preference, and treatment expectation were especially developed for use in this study and are not validated. Last, no statistical comparison was made between the results for the overall population and the Japanese subpopulation.

## Conclusions

The subgroup analysis of the SENSE study confirmed that Japanese patients with RA with moderate-to-high disease activity and poorly controlled disease were dissatisfied with their current RA treatment, mainly driven by dissatisfaction of treatment effectiveness. Despite the low level of satisfaction, they reported high self-perceived treatment adherence. The patients included in the current study had a high preference for an oral route of administration and expected a rapid onset of action with durable pain control and lasting relief of RA symptoms. In terms of risk acceptance, the highest level of acceptance was related to manageable side effects. A strong desire for educational support was also expressed in parallel with low digital health literacy. Our results also indicate that the perception and unmet needs of the current RA treatment in patients with moderate-to-high disease activity may differ by region or country. A tailored approach considering region- or country-specific situations may be warranted to consider the T2T approach in RA.

## Supporting information

S1 File Contains all the supporting S1-S4 Tables. (DOCX)

S2 File Protocol_H17-155 (SENSE) amendment. (PDF)

S3 File Figure source tables. (XLSX)

S1 Checklist TREND statement checklist. (DOCX)
