# Supplementary material for: Patient satisfaction, preferences, expectations, characteristics, and impact of suboptimal control of rheumatoid arthritis: A subgroup analysis of Japanese patients from a large international cohort study (SENSE)
Source: PLoS One. 2021 Nov 15;16(11):e0259389. doi: 10.1371/journal.pone.0259389 (PMC8592402; doi:10.1371/journal.pone.0259389)
Supplement: S1 File — (DOCX) [file pone.0259389.s001.docx]

**Supporting information**

**S1 Table. Dependent variables used in separate multivariate logistic regression models.**

| The following variables were used as dependent (i.e., response) variables in separate multivariable logistic regression models:  Model 1: Switching to a different DMARD planned (yes)  Model 2: Switching to a different DMARD planned (no)  Model 3: Switching to a tsDMARD planned (yes, no)  Model 4: Patient preference for oral administration (yes, no)  Model 5: Patient preference for monotherapy (yes, no)  Model 6: Good adherence (yes, no)  Model 7: Good treatment satisfaction (yes, no)” |
| --- |

DMARD, disease-modifying antirheumatic drug; tsDMARD, targeted synthetic DMARD.

**S2 Table. Current DMARD treatment (Japanese subpopulation).**

| **n=118** | | **n** | **Percentage of total (n=118)** | **Percentage of respective group** |
| --- | --- | --- | --- | --- |
| Current treatment with DMARDs |  | 117 | 99.2 | 100.0 |
|  | As monotherapy^a^ | 51 | 43.2 | 43.6 |
| Current treatment with tsDMARDs |  | 6 | 5.1 | 100 |
|  | As monotherapy | 1 | 0.8 | 16.7 |
| Current treatment with csDMARDs |  | 108 | 91.5 | 100 |
|  | As monotherapy | 42 | 35.6 | 38.9 |
| Current treatment with bDMARDs |  | 42 | 35.6 | 100 |
|  | As monotherapy | 8 | 6.8 | 19.0 |

^a^Monotherapy is defined as no concurrent administration of another DMARD, irrespective of other RA medications used.

bDMARD, biologic DMARD; csDMARD, conventional synthetic DMARD; DMARD, disease-modifying antirheumatic drug; RA, rheumatoid arthritis; tsDMARD, targeted synthetic DMARD.

**S3 Table. Current medications administered for RA (Japanese subpopulation).**

| **RA medication** | **Total** | |
| --- | --- | --- |
|  | **n** | **%** |
| Total | 118 | 100 |
| Methotrexate | 74 | 62.7 |
| Other | 74 | 62.7 |
| Sulfasalazine | 25 | 21.2 |
| Abatacept | 15 | 12.7 |
| Tocilizumab | 9 | 7.6 |
| Golimumab | 6 | 5.1 |
| Etanercept | 5 | 4.2 |
| Adalimumab | 3 | 2.5 |
| Baricitinib | 3 | 2.5 |
| Tofacitinib | 3 | 2.5 |
| Certolizumab pegol | 2 | 1.7 |
| Infliximab | 2 | 1.7 |
| Leflunomide | 1 | 0.8 |

RA, rheumatoid arthritis.

**S4 Table. Patient-reported outcomes (Japanese subpopulation).**

| **Variable** |  |
| --- | --- |
| FACIT-F (0-52)^a^, mean (SD) | 32.9 (10.4) |
| Duration of morning stiffness (hours)^b^, mean (SD) | 1.7 (3.5) |
| Worst joint pain (0-10, NRS)^a^, mean (SD) | 4.9 (2.7) |
| Self-reported adherence (0-100%, VAS; %)^a^, mean (SD) | 93.5 (13.8) |
| Good adherence (self-reported adherence, ≥80%), n (%)^a^ | 105 (89.7) |
| Poor eHealth Literacy score, n (%) | 75.0 (70.1) |

Data are presented as mean (SD) unless otherwise specified.

^a^n=117.

^b^n=94.

FACIT-F, Functional Assessment of Chronic Illness Therapy-Fatigue; SD, standard deviation; VAS, visual analog scale.
