# Supplementary material for: Patient satisfaction, preferences, expectations, characteristics, and impact of suboptimal control of rheumatoid arthritis: A subgroup analysis of Japanese patients from a large international cohort study (SENSE)
Source: PLoS One. 2021 Nov 15;16(11):e0259389. doi: 10.1371/journal.pone.0259389 (PMC8592402; doi:10.1371/journal.pone.0259389)
Supplement: S2 File — (PDF) [file pone.0259389.s002.pdf]

Cross-sectional Study Evaluating patieNt satisfaction, adherence featureS, and thEir association with socio-demographic and clinical characteristics of DMARD-inadequate responder rheumatoid arthritis patients - SENSE

Protocol H17-155

Amendment #01 18 MAR 2019

Title	Cross-sectional Study Evaluating patieNt satisfaction, adherence featureS, and thEir association with socio-demographic and clinical characteristics of DMARD-inadequate responder rheumatoid arthritis patients - SENSE
Date of Last Version of Protocol	18 Mar 2019
Sponsor	AbbVie

This study will be conducted in compliance with this protocol.

Confidential Information

No use or disclosure outside AbbVie is permitted without prior written authorization from AbbVie.

Table of Contents

1.0 Abbreviations 4
2.0 Responsible Parties 6
3.0 Abstract 7
4.0 Amendments 9
5.0 Milestones 9
6.0 Rationale and Background 9
6.1 Background 9
6.2 Rationale 11
7.0 Research Question and Objectives 11
7.1 Primary Objective 12
7.2 Secondary Objectives 12
8.0 Research Methods 13
8.1 Study Design 13
9.0 Setting 15
9.1 Variables 16
9.1.1 Disease activity measured by $DAS28_{CRP}$, CDAI and SDAI 16
9.1.2 Morning stiffness and Joint pain 17
9.1.3 HAQ-DI 18
9.1.4 FACIT-F version 4 18
9.1.5 TSQM version 1.4 18
9.1.6 Self-Reported Adherence (Appendix 2) 19
9.1.7 WPAI-RA 19
9.1.8 SF-36 version 2 20
9.1.9 eHealth Literacy Scale 20
9.1.10 Questionnaires to assess patient needs for support via PSP, medication preferences and treatment expectations (Appendices 3, 4, 5) 21
9.1.11 Health care resource utilization (HRU) (Appendix 6) 21
9.2 Data Sources 21
9.3 Study Size 22
9.4 Data Management 22
9.5 Data Analysis 23
9.5.1 Primary variable 23
9.5.2 Secondary variables 23
9.5.3 Data Analysis 24
9.6 Quality Control 26
9.7 Limitations of the Research Methods 26
9.8 Other Aspects 27

10.0 Protection of Human Subjects 27
11.0 Safety Reporting 27
11.1 Patient Reported Outcomes and/or Quality of Life Questionnaires.........28
11.1.1 Adverse Reaction Definition 28
11.1.2 Pregnancy Reporting 28
11.2 Product Complaint...28
11.2.1 Definition 28
11.2.2 Complaint Reporting 29
12.0 Country Information Page 30
13.0 Plans for Disseminating and Communicating Study Results32
14.0 References 32
15.0 Appendices 39
Appendix 1: List of copyright protected patient reported outcome questionnaires............39
Appendix 2: Self – reported adherence 40
Appendix 3: Questionnaire to assess patient needs for support via PSP 41
Appendix 4: Questionnaire to assess medication preferences...43
Appendix 5: Questionnaire to assess treatment expectations...44
Appendix 6: Health care resource utilization (HRU) in the past 3 months from enrollment45
Appendix 7: List of changes in Amendment #01 46

1.0 Abbreviations

ACR	American College of Rheumatology
ACPA	Anti-Citrullinated Peptide Antibody
AS	Ankylosing spondylitis
bDMARD	Biological Disease-Modifying Anti-Rheumatic Drug
csDMARD	Conventional Synthetic Disease-Modifying Anti-Rheumatic Drug
CDAI	Clinical Disease Activity Index
CI	Confidence Interval
CRF	Case Report Form
CRP	C-Reactive Protein
DAS28	Disease Activity Score, 28 joints
DMARD	Disease-Modifying Anti-Rheumatic Drug
DMARD-IR	Disease-Modifying Anti-Rheumatic Drug -Inadequate Responder
eCRF	Electronic Case Report Form
EGA	Evaluator (physician) Global Assessment of disease activity
eHEALS	Electronic Health Literacy Scale
ESR	Erythrocyte Sedimentation Rate
EULAR	European League Against Rheumatism
FACIT-F	Functional Assessment of Chronic Illness Therapy-Fatigue Scale
GCP	Good Clinical Practice
GEP	Good Epidemiological Practice
HAQ-DI	Health Assessment Questionnaire Disability Index
HCP	Health Care Professional
HRQOL	Health Related Quality of Life
HRU	Health care resource utilization
IL-6	Interleukin-6
IMID	Immune-mediated Inflammatory Disease
IR	Inadequate Responder
IV	Intravenous
JAK	Janus Kinase
MCS	Mental Component Score

MDGA	Medical Doctor Global Assessment (Physician Global Assessment)
mm	Millimeter
MTX	Methotrexate
MEDRA	Medical Dictionary for Regulatory Activities
NRS	Numerical Rating Scale
OR	Odds Ratio
PCS	Physical Component Score
PGA	Patient Global Assessment of disease activity
PMOS	Post-Marketing Observational Study
PRO	Patient Reported Outcome
PSP	Patient Support Program
RA	Rheumatoid arthritis
RF	Rheumatoid Factor
SAP	Statistical Analysis Plan
SDAI	Simple Disease Activity Index
SC	Subcutaneous
SF-36v2	36-Item Short Form Health Survey, Version 2.0
SJC	Swollen Joint Count
TAI	Total Activity Impairment
TJC	Tender Joint Count
TNF	Tumor Necrosis Factor
TNFi	Tumor Necrosis Factor Inhibitor
TSQM	Treatment Satisfaction Questionnaire for Medication
tsDMARD	Targeted Synthetic Disease-Modifying Anti-Rheumatic Drug
TWPI	Total Work Productivity Impairment
UC	Ulcerative Colitis
VAS	Visual Analogue Scale
WPAI	Work Productivity and Activity Impairment
WPAI-RA	Work Productivity and Activity Impairment, Rheumatoid Arthritis
WPAI-GH	Work Productivity and Activity Impairment, General Health
WPAI-SHP	Work Productivity and Activity Impairment, Specific Health Problem

2.0 Responsible Parties

Study Designated Physician (SDP) and Scientific Lead:

Orsolya Nagy MD, PhD, Rheumatology Lead for EEMEA
AbbVie Ltd., Lechner Ödön fasor 7, 1095 Budapest, Hungary
Phone: + 36 30 635 0999; e-mail: orsolya.nagy@abbvie.com

Protocol Authors:

Orsolya Nagy MD, PhD, EEMEA Rheumatology Medical Lead
AbbVie Ltd., Lechner Ödön fasor 7, 1095 Budapest, Hungary
Phone: + 36 30 635 0999; e-mail: orsolya.nagy@abbvie.com

Kun Chen, PhD, Associate Director
Data and Statistical Science, R&D Statistics,
GPRD DSS, Bldg AP09A-LL, 1 North Waukegan Road, North Chicago, IL 60064
Phone: +1 847-935-8533, e-mail: kun.chen@abbvie.com

Biostatistics and Data Management:

Biostatistics: Dr. Michael Obermeier
GKM Gesellschaft für Therapieforschung GmbH
Lessingstrasse 14; D-80336 München, Germany
Phone: +49(89)209120-815 · Fax: +49(89)209120-30
e-mail: m.obermeier@gkm-therapieforschung.de
www.gkm-therapieforschung.de

Data Management: Daniela Domler
GKM Gesellschaft für Therapieforschung GmbH
Lessingstrasse 14; D-80336 München, Germany
Phone: +49(89)209120-56 · Fax: +49(89)209120-30
e-mail: d.domler@gkm-therapieforschung.de
www.gkm-therapieforschung.de

3.0 Abstract

Title: Cross-sectional Study Evaluating patieNt satisfaction, adherence featureS, and thEir association with socio-demographic and clinical characteristics of DMARD-inadequate responder rheumatoid arthritis patients - SENSE

Background

Rheumatoid arthritis (RA), if not controlled properly, may result in severe and progressive articular damage, loss of function, deterioration of the quality of life, and increased mortality. Despite the wide range of treatment options, many patients with RA remain sub-optimally managed and sustained remission is rarely achieved.

Low adherence rate is a serious medical problem in RA and represents a significant financial burden to health care and society. Suboptimal disease control in RA could be a result of low medication adherence. Satisfaction with medication attributes has been found to affect patients' dosing adherence and persistence with treatment over time.

Although patient satisfaction is a useful and essential outcome to assess, patient satisfaction in patients not responding to their current therapies is not well examined. Identifying areas lacking satisfaction in the group of patients with inadequate treatment response, thus candidates for treatment regimen modification, can guide the future treatment decision. In addition to sub-optimal RA control assessed by traditional clinical measures, patients' needs remain frequently unmet across key patient reported outcome (PRO) domains such as pain, physical function, mental function, and fatigue, which can all adversely affect their adherence, social function, sexual function, the ability to work, and overall well-being.

Rationale

There is a need for better and in-depth understanding the clinical, socio-demographic, health-economic, adherence and PRO characteristics of RA patients with inadequate response to currently available DMARDs. Moreover, in the same sub-optimally controlled RA population, additional data are necessary about their treatment satisfaction, expectations and treatment preferences for RA. Evaluating differences in the above listed areas across subgroups of patients treated with different treatment modalities and regimes, route of RA treatment administration, clinical, demographic and PRO characteristics can guide personalized treatment, support shared treatment decision making and help achieve better disease outcomes in RA. Data collected in the study can also support the development of effective tools for improving adherence and may inform the optimal design of future patient support programs. Results will also inform about the current treatment strategies used in the management of the sub optimally controlled RA population.

Research Question and Objectives:

Primary Objective: To assess treatment satisfaction of patients with sub optimally controlled RA treated with conventional/targeted synthetic or biological DMARDs.

Definition of sub optimally controlled RA: moderate to high disease activity as measured by DAS28.

Secondary Objectives:

- To assess sociodemographic, clinical, functional, adherence and quality of life characteristics of sub optimally controlled RA patients treated with conventional/targeted synthetic or biological DMARDs.
- To assess healthcare resource utilization during 12 months prior to enrollment to the study in sub optimally controlled RA patients treated with conventional/targeted synthetic or biological DMARDs.
- To assess electronic health literacy of sub optimally controlled RA patients treated with conventional/targeted synthetic or biological DMARDs.
- To assess expectations towards RA therapy, medication preferences and needs for patient support of sub optimally controlled RA patients treated with conventional/targeted synthetic or biological DMARDs
- To determine relationship between treatment satisfaction, its subdomains and patient characteristics.
- To determine relationship between adherence and patient characteristics.
- To determine relationship between medication preferences and patient characteristics.
- To determine relationship between treatment expectations and patient characteristics.
- To determine relationship between specific PSP-related needs and patient characteristics.
- To evaluate the above measures in various subgroups of patients stratified by treatment modalities used, by clinical characteristics, by presence of comorbidities, if there are sufficient patients in each subgroup.

To understand treating rheumatologists treatment strategy in the study population and its relationship with patient characteristics.

**Study Design:** Multi-country, multicenter, cross-sectional non-interventional study.
**Data Analysis:**<br>Data from all clinical assessments of the patients will be summarized using summary statistics. Additional exploratory analyses may be added on a data driven basis and will be specified in SAP. Separate analysis is planned for each country based on the needs of the particular participating country.<br>Statistical analysis in general will include: descriptive statistics, comparative subgroup analysis, relationship between variables and predictive models. Detailed statistical analysis plan (SAP) will be provided separately.

4.0 Amendments

Number	Date	Section of Study Protocol	Amendment or Update	Reason
1	*18 March 2019*	*5.0*<br>*9.1*<br>*9.3*<br>*Appendix 1*	*Amendment*	• *Modify study milestones*<br>• *Removal of copyright protected PRO questionnaires from the Appendix*<br>• *Modify sample size*<br>• *Correct typos*<br><br>*See Appendix 7 for details*

5.0 Milestones

Major study milestones and their planned dates are as follows:

Start of Data Collection:	02 September 2018
End of Data Collection:	31 May 2019
Final Report of Study Results:	01 December 2019

6.0 Rationale and Background

6.1 Background

Rheumatoid arthritis (RA) is a chronic inflammatory disease which, if not controlled properly, may result in severe and progressive articular injury, loss of function, deterioration of the quality of life, and increased mortality [1-4]. Biological disease modifying drugs (bDMARDs) are typically used as second-line therapy in patients with an inadequate response to conventional synthetic disease modifying drugs (csDMARDs). The group of targeted synthetic DMARDs (tsDMARDs) represents a novel therapeutic option for patients with inadequate response to cs/bDMARDs [13-22]. However, the role of tsDMARDs in current treatment paradigm is not yet fully established, partially since we still have limited data on their real-word effectiveness and on the factors associated with sub-optimal response to them [23].

Despite the fact that the range of treatment options is steadily increasing and RA therapies help to slow down disease progression and the development of joint damage, many patients with RA remain sub-optimally managed with sustained remission rarely achieved [5-11]. Two-thirds of RA patients do not respond adequately to their initial bDMARD, and sustained clinical remission is achieved in only 20–40% of patients treated with DMARDs [5, 6]. Moreover, radiographic progression can still occur even in the presence of clinical remission [12]. Suboptimal management of RA results in increased healthcare resource use and medical costs [9].

Treatment adherence is of particular importance in RA as it is characterized by fluctuating and usually progressive disease courses and a need for lifetime management [27-32]. Despite the fact that the efficacy of available medications is proven even with long term use [37-39], the level of adherence is still suboptimal among RA patients, varying from 30 to 80% [4,33], thus resulting in suboptimal outcomes and representing a significant financial burden to health care [34-36,42-43]. There is evidence available confirming that bDMARD use is associated with better adherence than conventional oral DMARDs in RA [40].

Patients' satisfaction with therapy is associated with the level adherence and treatment choice [44]. Measuring satisfaction can cover various areas, e.g. evaluation of the acceptability of care or treatments; comparison of health-care programs or treatment options; identification of service or treatment approaches that require change; and screening of patients who are likely to become non-adherent to care plans or medication regimens [44]. Therefore, patient satisfaction is a useful outcome measure for evaluating the benefit of patient support programs, services and products, and can inform development of products, services and devices [45-47, 51-54]. Satisfaction is also closely linked to patients' **treatment expectations** mainly related to their everyday life, personal concerns, level of pain, and sometimes there is a disconnection between clinicians' and patient's treatment goals [52]. In order to have better adherence and disease outcomes, treating physicians have to better understand patient expectations [50, 53, 54].

Identifying areas lacking satisfaction (e.g. related to convenience or tolerability) in the group of RA patients with inadequate treatment response, thus candidates for treatment regimen modification, can guide the future treatment decision. The subsequent treatment regimen based on the identified areas with low satisfaction, in line with patient preferences will be more likely associated with higher willingness to adhere to prescription, which will ultimately lead to higher real-life efficacy [39].

As shown by a recently published literature review [78], despite the wide array of available treatments for RA, clinical and patients' needs remain unmet across key patient related outcome (PRO) domains such as pain, physical function, mental function, and fatigue, which can all adversely affect adherence, social function, sexual function, the ability to work, and overall well-being [79]. This supports the trend for more patient-centered care, better doctor–patient communication and shared decision-making [54-59], ultimately improving the quality of care, as an overarching principle recommended by EULAR [23]. Among PROs, **fatigue** in RA is of particular interest [60] since it has been consistently shown to be important for patients with RA [61], having consequences on all aspects of quality of life [62]. Results from independent studies highlight that **joint stiffness** is also an important feature of RA [63], and its level reflect functional disability and pain more than traditional markers of inflammation [64]. Similarly, people with RA identify **joint pain** as their most important symptom, one that often persists despite optimal control of inflammatory disease [52, 65], thus non-inflammatory components of RA pain should be considered when gauging eligibility for or response to RA therapies [65].

6.2 Rationale

There is a need for better and in-depth understanding the clinical, socio-demographic, health-economic, adherence and PRO characteristics of RA patients with inadequate response to currently available DMARDs.

Moreover, in the same sub-optimally controlled RA population, additional data are necessary about their treatment satisfaction, expectations and treatment preferences for RA.

Evaluating differences in the above listed areas across subgroups of patients treated with different treatment modalities and regimes, route of RA treatment administration, clinical, demographic and PRO characteristics can guide personalized treatment, support shared treatment decision making and help achieve better disease outcomes in RA.

Data collected in the study can also support the development of effective tools for improving adherence and may inform the optimal design of future patient support programs.

Results will also inform about the current treatment strategies used in the management of the sub optimally controlled RA population.

7.0 Research Question and Objectives

The research question is as follows: what are the clinical, socio-demographic, workability, healthcare resource utilization characteristics, treatment satisfaction, preferences and residual unmet needs of

patients with inadequate response to their current RA treatment with conventional/targeted synthetic or biological DMARDs?

7.1 Primary Objective

The primary objective of the study is to assess treatment satisfaction of patients with sub optimally controlled RA, treated with conventional/targeted synthetic or biological DMARDs.
Definition of sub optimally controlled RA: moderate to high disease activity as measured by DAS28.

7.2 Secondary Objectives

- To assess the sociodemographic, clinical, functional, adherence and quality of life characteristics of sub optimally controlled RA patients treated with conventional/targeted synthetic or biological DMARDs.
- To assess healthcare resource utilization (HRU) during 12 months prior to enrollment to the study in sub optimally controlled RA patients treated with conventional/targeted synthetic or biological DMARDs.
- To assess electronic health literacy of sub optimally controlled RA patients treated with conventional/targeted synthetic or biological DMARDs.
- To assess expectations towards RA therapy, medication preferences and needs for patient support of sub optimally controlled RA patients treated with conventional/targeted synthetic or biological DMARDs
- To determine relationship between treatment satisfaction, its subdomains and patient characteristics.
- To determine relationship between adherence and patient characteristics.
- To determine relationship between medication preferences and patient characteristics.
- To determine relationship between treatment expectations and patient characteristics.
- To determine relationship between specific PSP-related needs and patient characteristics.
- To evaluate subgroups of patients, if there are sufficient patients in each subgroups:

- To evaluate the above measures in the subgroups of patients treated with different treatment modalities, regimes and line therapy and route of administration (e.g. patients treated with csDMARDs, bDMARDs, tsDMARDs; patients treated with orally or parenterally administered DMARDs (SC or IV), patients treated in monotherapy, patients treated with combination therapy, patients treated with first or second line bDMARD).
- To evaluate the above measures in subgroups of patients with different clinical characteristics (e.g. with high or moderate disease activity).
- To evaluate the above measures between the subgroups of patients with and without comorbidities including cardiovascular comorbidities.

- To understand treating rheumatologists' treatment strategy in the study population and its relationship with patient characteristics.

8.0 Research Methods

8.1 Study Design

This is a multi-country, multi-center, cross-sectional, non-interventional study. The data collection period will be approximately 9 months. Each patient attends a single study visit.

The following data will be collected:

1. Demographic data: age, gender, race, occupation, education, place of living (rural/urban)

2. RA disease related data

- Year of RA diagnosis
- Diagnostic criteria used for RA diagnosis
- Current disease activity measured by DAS28 and SDAI/CDAI
- Imaging results: Presence/absence of structural damage by X-ray (X-ray assessment not older than 6 months from enrollment is acceptable)
- Laboratory tests: rheumatoid factor (RF) (not older than 3 months from enrollment), anti-citrullinated protein antibody (ACPA) (not older than 3 months from enrollment), current CRP

- Current co-morbidities (including but not restricted to metabolic, cardiovascular, mental, endocrine diseases according latest version of MeDRA)

3. Medication related data

- Prior and current treatment for RA
- Current concomitant medication for comorbidities
- Treatment strategy of treating rheumatologists: whether there is a plan for switching to a different DMARD (Yes / No); in case of plan to switch: mode of action of next DMARD.

4. Health care resource utilization (HRU) in the past 3 months from enrollment.

5. Patient reported outcomes

- Current physical function measured by HAQ-DI
- Level of fatigue measured by FACIT-F
- Duration and severity of morning stiffness measured by VAS
- Level of worst joint pain measured by VAS
- Satisfaction with current treatment for RA measured by TSQM version 1.4
- Self-reported adherence measured by 0-100 mm VAS
- Workability measured by WPAI-RA
- Quality of life health assessment measured by SF-36v2
- Assessments on electronic health literacy by eHEALS
- Questionnaires to assess patient needs for support via PSP, medication preferences and treatment expectations

9.0 Setting

As this is an observational, non-interventional study, patient's treatments are determined solely by the treating physician, which falls within the scope of the physician's/institution's general liability insurance coverage and precedes the decision to offer the patient the opportunity to participate in the study.

According to the requirements for non-interventional or observational studies, no additional diagnostic or monitoring procedures will be applied to the patients included in the study other than those which would ordinarily be applied in the course of the particular therapeutic strategy.

Site and Researcher Selection

The research sites will be community or hospital based medical centers experienced in the treatment of RA.

Researchers will have access to RA patient population and have the ability to appropriately conduct the study in accordance with applicable legal and regulatory requirements.

Patient Selection Criteria

Patient attending a routine visit, who fulfill ALL the following selection criteria, can be included:

- Male or female.
- Adult (aged ≥ 18 years).
- Has rheumatoid arthritis (RA), diagnosed either by the 1987-revised ACR classification criteria or by the 2010 ACR / EULAR classification criteria for RA.
- Currently treated with any kind of approved csDMARDs, tsDMARDs or bDMARDs.
- Has been exposed to no more than 2 bDMARDs at the time of the enrollment.
- His/her RA is sub optimally controlled, despite full tolerable dose of current DMARD therapy administered for ≥3 months.

  - Definition of suboptimal disease control: having high or moderate disease activity (as defined by having DAS28 > 3.2) for at least 1 month but not more than for 4 months prior to the enrollment.

- Understands the language and willing/able to complete the patient reported outcome questionnaires.
- Does not participate in any kind of clinical study for RA.
- Has provided written authorization to the investigator to use and/or disclose personal and/or health data, or informed consent if requested by the local regulations.

9.1 Variables

Copyright protected outcome measures and patient questionnaires are listed in Appendix 1.

9.1.1 Disease activity measured by $DAS28_{CRP}$, CDAI and SDAI

Composite indices or pooled indices are useful tools for the evaluation of disease activity in patients with rheumatoid arthritis (RA). They allow the integration of various aspects of the disease into a single numerical value, and may therefore facilitate consistent patient care and improve patient compliance, which both can lead to improved outcomes. The DAS score [66] measures a patient's level of disease activity at a given time using tender joint count (TJC), swollen joint count (SJC) across 44 joints, ESR or CRP and patient's health assessment along a VAS (0-100 mm).

The DAS28 score is a modified version that is scored across a reduced set of 28 joints, omitting the feet. As Disease Activity Score 28-joint count (DAS28) is currently the most commonly used composite measure of RA disease activity in clinical practice, remission and low disease activity will be defined as follows [67]:

- Remission if DAS28 score < 2.6.
- Low disease activity (LDAS) if DAS28 score $\leq$ 3.2.
- Moderate disease activity if DAS28 score is > 3.2 -5.1.
- High disease activity if DAS28 score is > 5.1.

In this study, $DAS28_{CRP}$ is preferred since it is frequently used in clinical research, due to several reasons. Firstly, CRP measurements are routinely used in clinical practice, and are often available in

circumstances when ESR measurements are not. CRP levels are more sensitive to short-term changes in disease activity [68], whereas ESR can be influenced by a number of unrelated factors, such as age, gender or plasma proteins. Lastly, laboratory tests used to calculate CRP are faster than those used to measure ESR, and measurements can be standardized in a central laboratory for multicenter clinical trials. Due to variability of practice across participating countries, $DAS28_{ESR}$ will be accepted.

The Simplified Disease Activity Index (SDAI) and the Clinical Disease Activity Index (CDAI) [69, 70] are two new tools for the evaluation of disease activity in RA. They have been developed to provide physicians and patients with simple and more comprehensible instruments. Moreover, the CDAI is the only composite index that does not incorporate an acute phase response and can therefore be used to conduct a disease activity evaluation essentially anytime and anywhere. These two new tools have not been developed to replace currently available instruments such as the DAS28, but rather to provide options for different environments.

Cut-points of SDAI:

- High disease activity > 26
- Moderate disease activity > 11 - 26
- Low disease activity > 3.3 - 11
- Remission ≤ 3.3

Cut-points of CDAI

- High disease activity > 22
- Moderate disease activity > 10 - 22
- Low disease activity ≥ 2.8 - 10
- Remission ≤ 2.8

9.1.2 Morning stiffness and Joint pain

Although morning stiffness is not specific to RA, changes in morning stiffness for an individual patient are helpful when monitoring health status. Health professionals can ask about morning stiffness but the

most accurate and consistent approach to assessment from one visit to the next appears to be a patient self-report questionnaire.

In this study, a visual analogue scale using numeric rating scale (NRS) will be used to assess severity and length of morning stiffness in the past 7 days. Similar NRS will be employed to assess the worst joint pain in the past 7 days prior to the study.

9.1.3 HAQ-DI

Health Assessment Questionnaire (HAQ) is a widely accepted, validated, rheumatology specific instrument to assess physical function in RA [71]. It consists of 20 questions, covering eight types of activities. For each question, scores range from 0 to 3 (0 = without any difficulty; 1 = with some difficulty; 2 = with much difficulty or with assistance; 3 = unable to do). HAQ Disability Index (HAQ-DI) score is the average of the highest score in each of the eight categories.

9.1.4 FACIT-F version 4

Although there are several validated tools for the measurement of fatigue, there is no gold standard. One self-report questionnaire that has been validated for use with older adults is the Functional Assessment of Chronic Illness Therapy (FACIT) Fatigue Scale (Version 4). The FACIT Fatigue Scale is a short, 13-item, easy to administer tool that measures an individual's level of fatigue during their usual daily activities over the past week. The level of fatigue is measured on a four point Likert scale (0 = not at all fatigued to 4 = very much fatigued) [72]. The FACIT Fatigue Scale is one of many different FACIT scales that are part of a collection of health-related quality of life (HRQOL) questionnaires targeted to the management of chronic illness referred to as The FACIT Measurement System. The group tests newly constructed FACIT subscales on a sample of at least 50 subjects. The FACIT tool has been translated in more than 45 different languages permitting cross-cultural comparisons.

9.1.5 TSQM version 1.4

Although numerous disease-specific measures of patients' treatment satisfaction for medication have been reported in the literature, less attention has been paid to developing a more general measure of TSQM one that would permit comparisons across medication types and patient conditions. To fill this gap, the TSQM was developed [51, 73]. TSQM is a generic measure of treatment satisfaction for medication, was rigorously developed with sound psychometric properties. Multiple linguistically

validated languages available. Three versions are available: **TSQM Ver 1.4** (14 items), TSQM Ver II (11 items), and TSQM Ver 9 (9 items).

Domains include: Effectiveness, Side effects, Convenience, Global Satisfaction. This measure attempts to show that adherence is expected to be related with patients' satisfaction with therapy and such satisfaction can be a function of not only the effect of the treatment, but also the services offered. In the study, TSQM-1.4 will be employed.

9.1.6 Self-Reported Adherence (Appendix 2)

The terms "adherence" and "compliance" are often used interchangeably, however adherence is becoming the preferred term as it suggests the patient has a less passive role in the management of their medication [34].

In this study a 0-100 mm VAS will be employed to gain numeric insights about patients' estimation of drug intake adherence.

Visual analog scales (VASs) have been extensively used for assessment in a number of health domains (e.g., acute and chronic pain) and have recently been applied to assessment of antiretroviral therapy medication adherence [74]. It has been shown that patients' self-evaluation of drug intake evaluated by VAS scores correlates with objective adherence measures and thus can be a valuable, easy to use tool for assessing adherence in IMIDs in routine clinical practice [40, 74, 84].

9.1.7 WPAI-RA

The Work Productivity and Activity Impairment (WPAI) Questionnaire [75] was created as a patient-reported quantitative assessment of the amount of absenteeism, presenteeism and daily activity impairment attributable to general health (WPAI-GH) or a specific health problem, such as RA (**WPAI-SHP, WPAI-RA**). The WPAI-GH and the WPAI-SHP were created simultaneously and use the same template, but in the GH version the subject is instructed to respond with reference to the general health status while in the SHP version the subject responds with reference to a specified health problem, disease (RA) or condition. The following 4 outcomes will be expressed as a percentage from 0 to 100:

- % Presenteeism – percentage of impairment while working due to the respective IMID.
- % Absenteeism – percentage of work time missed due to the respective IMID.

- % Total work productivity impairment (TWPI) – percentage of overall work impairment due to the respective IMID

- % Total activity impairment (TAI) – percentage of general (non-work) activity impairment due to the respective IMID.

The percentage of work time missed (absenteeism) is calculated as Q2/ (Q2 + Q4) x 100%. The percentage of impairment while working (i.e. presenteeism) is calculated as Q5/10 x 100%. The percentage of total work productivity impairment ((TPI) i.e., work productivity loss) is Q2/ (Q2 + Q4) + (1–Q2/ (Q2+Q4) x (Q5/10) x 100%. The percentage of total activity impairment (TAI) is calculated as Q6/10 x 100%.

9.1.8 SF-36 version 2

Sort Form -36 version 2 is a multi-purpose, short-form health survey with only 36 questions [76]. It yields an 8-scale profile of functional health and well-being scores as well as psychometrically-based physical and mental health summary measures and a preference-based health utility index. It is a generic measure, as opposed to one that targets a specific age, disease, or treatment group. Accordingly, the SF-36 has proven useful in surveys of general and specific populations, comparing the relative burden of diseases, and in differentiating the health benefits produced by a wide range of different treatments. In 1996, Version 2.0 of the SF-36 (SF-36v2) was introduced, to correct deficiencies identified in the original version. Those improvements were implemented after study using both qualitative and quantitative methods. The SF-36v2 has 2 summary scores, the Physical Component Summary (PCS) and Mental Component Summary (MCS) scores, and 8 domain scores: physical function, bodily pain, role limitations–physical, general health, vitality, social function, role limitations–emotional, and mental health. Domain scores range from 0 to 100, with greater scores reflecting better health status. The SF-36v2 summary and domain scores have excellent reliability and good construct validity across the general population as well as chronic disease populations including RA.

9.1.9 eHealth Literacy Scale

The eHealth Literacy Scale (eHEALS) has been developed to address the need to assess eHealth literacy for a wide range of populations and contexts [41]. The eHEALS is a self-report tool that can be administered by a health professional and is based on an individual’s perception of her or his own skills

and knowledge within each measured domain. The instrument is designed to provide a general estimate of consumer eHealth-related skills that can be used to inform clinical decision making and health promotion planning with individuals or specific populations.

9.1.10 Questionnaires to assess patient needs for support via PSP, medication preferences and treatment expectations (Appendices 3, 4, 5)

The questionnaires being used in this study are developed by AbbVie based on the available literature and patient research [39, 48, 53, 77-79] due to limited availability of appropriate validated tools.

9.1.11 Health care resource utilization (HRU) (Appendix 6)

Health resource utilization 12 months preceding the study inclusion will be assessed as follows:

- Number of visits by a health care professional for RA in the past 3 months.
- Number and length of hospitalizations in the past 3 months.
- Number of emergency visits in the past 3 months.

To obtain 12-month health care resource utilization and sick leave before the enrollment, the collected 3-month data will be multiplied by 4. The rationale for this approach is that a similar percentage of patients would consume these quantities of resources in any given 3-month period, and it avoids memory bias [80-82].

9.2 Data Sources

Data for this study will be collected during a single visit previously scheduled for a routine follow-up of the RA disease.

Data for relevant medical history will be obtained from patient charts and/or documentation.

Local language patient questionnaires will be used for assessing various PROs, treatment expectations, patient needs and preferences. Questionnaires listed in Appendices indicate the version used for the validated translations; specific country versioning may vary depending on the translation process.

Health care resource utilization (HRU) will be assessed using patient documentation and patient interview.

Only data which are part of routine will be collected, except PRO questionnaires which are not deemed as an intervention and are inherent part of epidemiological studies.

9.3 Study Size

Approximately 1500 RA patients will be included in the study. The study will include 100 sites in approximately 20 countries in Europe, America and Asia. Countries will recruit between 30 and 200 patients per country depending on the available patient population.

Current sample size calculation is based on information of global satisfaction measured by TSQM 1.4. It is also assumed that similar number of patients will be allocated to each country so that country level analyses will be explored, as well as overall population. Based on the literature [51, 83], it seems reasonable to assume that the standard deviation for the global satisfaction is about 20 with mean value range from 71 – 91.

A given sample size of N=1500 will be able to provide a 95% confidence interval (CI) with a half width of 1.01 in the overall study population.

For country specific analysis a sample of N=50 will be able to provide a 95% confidence interval (CI) with a half width of 5.68. . For country specific analysis a sample of N=30 – 200 will be able to provide a 95% confidence interval (CI) with a half width of 7.47 to 2.79.

9.4 Data Management

Each center will document patient data in electronic case report forms (eCRF).

Diagnostic measures and observations routinely performed in patients included in this study will be entered by the researcher or staff under his/her supervision into the eCRFs provided by AbbVie, according to the protocol.

Only data specified in the protocol will be submitted to AbbVie.

The eCRF for investigators' use will be in English and will include the PROs and other questionnaires in English. Patients will receive questionnaires in their local language for self-administration. Results of patient completed questionnaires will be transcribed into the eCRF. Comorbidities will be coded according to up-to date version of MEDRA.

The investigator or delegated staff must complete the eCRFs. Neither AbbVie nor any agents acting on behalf of AbbVie may complete the eCRFs.

9.5 Data Analysis

9.5.1 Primary variable

The primary endpoint is the total satisfaction score as measured by TSQM 1.4 for the full study population.

9.5.2 Secondary variables

The secondary variables are:

- Sociodemographic, medication and disease characteristics including RA disease activity scores of DAS28, CDAI/SDAI and proportion of patients with high or moderate disease activity as per DAS28, CDAI/ SDAI.
- Proportion of patients in which treating physician is going to switch the DMARD therapy. Proportion of different mode of action of next planned DMARD.
- Effectiveness, Side effects, Convenience, Global Satisfaction sub scores of TSQM 1.4.
- Total fatigue score measured by FACIT-F.
- RA functional capacity score of HAQ-DI.
- Duration and severity of morning stiffness scores.
- Worst reported pain score.
- Self-reported adherence score (VAS).
- Total score and individual components of WPAI-RA score (% Presenteeism, % Absenteeism, % Total work productivity impairment, % Total activity impairment).
- General quality of life Total score, PCS-score and MCS-score domains and scale scores of SF-36v2 for the full study population.
- Total Electronic health literacy scale score as measured by eHEALS for the full study population.

- Health resource utilization assessment scores in 12 months preceding the study inclusion for the full study population.
- Proportion of patients with different level of patients' need of PSP support for each question point as measured by AbbVie developed questionnaire
- Proportion of patients for each question point about patients' medication preferences as measured by AbbVie developed questionnaire
- Proportion of patients with different level of patients' treatment expectations for each question point as measured by AbbVie developed questionnaire

9.5.3 Data Analysis

Data from all clinical assessments of the patients will be summarized using summary statistics. Detailed statistical analysis plan (SAP) will be provided separately. Additional exploratory analyses may be added on a data driven basis and will be specified in SAP. Separate analysis is planned for each country based on the needs of the particular participating country.

Statistical analysis in general will include:

Descriptive statistics

Continuous variables will be summarized with number of patients, mean, standard deviation, median, 1st quartile, 3rd quartile, minimum and maximum values, and 95% confidence interval (CI) for mean or median (as appropriate). Categorical variables will be summarized with the number and proportion of patients in each category. Appropriate CI for proportion will be provided. Visualization tools will be used to present the distribution of data, if applicable.

Comparative subgroup analysis

Subgroup analyses of all measures listed above are planned if there are sufficient patients in each subgroup listed below:

- According to the RA treatment
    - Patients currently treated with any synthetic DMARDs, tsDMARDs, csDMARDs, bDMARDs

  - Patients with previous exposure to 1 bDMARD or 2 bDMARD
  - Patients treated with orally or parenterally administered DMARD
  - Patients treated with IV or SC administered DMARD
  - Patients treated with DMARD monotherapy or combination therapy
- According disease activity (high or moderate)
- According to comorbidities
  - Patients with or without any kind of comorbidity
  - Patients with cardiovascular comorbidity

For continuous variables two sample t-tests or non-parametric tests will be used based on the data distribution; for categorical variables appropriate R×C tables will be constructed and Chi-square or exact Fisher tests will be used based on appropriate assumption.

Relationship between variables and predictive models

Relationship analysis is planned between treatment satisfaction and its subdomains; between adherence and its subdomains; between medication preferences, between treatment expectations and between specific PSP-related needs and patient characteristics in the full population and the subgroups specified above. Appropriate data visualization techniques will be used for relationships investigation. Correlation analysis for continuous variables or calculation of crude measure of effect for categorical data can be used if appropriate. Strength of relationships between two variables will be investigated with appropriate univariate regression analysis. Linear regression model will be used for continuous response and predictor variables that satisfy appropriate assumptions of conducting linear regression; logistic regression model will be used for in case of binary dependent variables and continuous or categorical independent variables that satisfy appropriate assumptions.

Predictive models will be constructed using the multivariate linear or logistic regression analyses, as appropriate to data type and regression assumptions. Stepwise selection will be used in defining estimation parameters of the final model. Predictors of switching DMARD therapy will be determined. Patient demographics and clinical characteristics will be considered in the multivariate analysis as predictor variables.

9.6 Quality Control

Oversight of the study is the responsibility of AbbVie.

The sites will be instructed in the protocol regarding the use of the questionnaires, the functionality and handling of the eCRF, and the requirements to maintain source documents for each patient in the study in order to ensure that all patient questionnaires will be completed by patients.

Different quality assurance methods will be implemented to ensure integrity of the information reported over the course of the project. All data will be entered via eCRF. The eCRFs will be submitted to be entered into the electronic database maintained by external data management provider.

After data entry, computer logic checks will be run to check for inconsistent data. Any necessary corrections will be made to the database and documented via queries, source data clarification forms and an audit trail regarding all changes will be available. A manual review of selected line listings will also be performed at the end of the study.

9.7 Limitations of the Research Methods

Results cannot be generalized to the whole RA patient population universe due to enrolment of sub optimally controlled patients.

Results must be carefully interpreted due to the observational and cross-sectional nature of the study. Information about the percentage of screened patients that agreed to participate in this study will be not collected which may result in selection bias.

No hypothesis testing will be performed in the primary analysis. All other statistical tests performed in secondary analysis will be performed without formal hypothesis testing and resulting p-values will be interpreted in a descriptive manner.

Factors associated to cultural differences may influence the results per countries. Country specific sub analyses should be interpreted carefully due to low sample size.

There is a potential imbalance in the size in subgroups per treatment used or per presence of comorbidities. Comparisons between different subgroups should be interpreted in exploratory manner only. Confounding factors (e.g., disease duration and severity, level of treatment response, and number and type of co-morbidities) may not be equally distributed between the groups, which can lead to bias and subsequent misinterpretation.

No validated questionnaires will be used for assessing needs for PSP, treatment preferences and expectations. Adherence results will be based on self-reported outcomes that may be influenced by self-presentational and recall biases. Patients may overestimate the extent of their adherence. Past HRU is based on patient interviews in addition to patient charts, similarly might result in recall-biases.

9.8 Other Aspects

The guidelines for good pharmacoepidemiology practices (GPP) in non-interventional studies will be respected and any applicable local laws and regulations. This trial is not in the scope of Good Clinical Practice (GCP) studies.

10.0 Protection of Human Subjects

This study will be run in compliance with local laws and regulations. Notification/submission to the responsible Ethics Committee, Health Institutions and/or Competent Authorities will be performed as required by local laws and regulations (see Country Information page [Section 12.0]).

Written authorization and/or informed consent will be obtained prior to patient inclusion as requested by local regulations.

11.0 Safety Reporting

Product-Related Events Including Adverse Reaction Reporting

This non-interventional study is not designed to identify or quantify a safety hazard relating to an AbbVie authorized product.

If a patient reports a product-related event (e.g., suspected adverse reaction or product complaint) to his/her healthcare professional during the data collection period or the healthcare professional identifies a product-related event, which is considered related to any AbbVie authorized product, the event should be reported to AbbVie. Any product-related events considered to be related to a non-AbbVie product should be reported in accordance with local laws and regulations to the relevant Regulatory Authority and/or drug marketing authorization holder.

The AbbVie contact details are specified in Country Information page (Section 12.0).

The data on medical history will be collected in a retrospective manner. This retrospective portion of the study is based on secondary use of data previously collected from healthcare professionals for other purposes. Any suspected adverse reactions (adverse events considered to be likely related to an AbbVie product) identified during the course of the retrospective review of data should be reported to AbbVie clearly specifying the suspected adverse reaction was identified during retrospective review in an AbbVie non-interventional study. Any suspected adverse reactions considered to be related to a non-AbbVie product should be reported in accordance with local laws and regulations to the relevant Regulatory Authority and/or drug marketing authorization holder.

11.1 Patient Reported Outcomes and/or Quality of Life Questionnaires

Patient Reported Outcome (PRO) and patient questionnaires data are not considered a potential source of adverse events for the purposes of this study. However, the Health Care Professional (HCP) should review the PRO or questionnaires data. If the HCP identifies a product-related event (such as a suspected adverse reaction), and if determined to be related to an AbbVie authorized product, report to AbbVie.

11.1.1 Adverse Reaction Definition

An adverse reaction is defined as any untoward medical occurrence in a patient, where there is evidence to suggest a causal relationship between the product and the adverse event.

11.1.2 Pregnancy Reporting

In the event of a pregnancy occurrence in a patient or partner taking any AbbVie authorized product during the course of the study, the researcher will report the pregnancy to AbbVie. Any pregnancy in a patient taking a non-AbbVie product should be reported in accordance with local laws and regulations to the relevant Regulatory Authority and/or drug marketing authorization holder.

The AbbVie contact details are specified in Country Information page (Section 12.0).

11.2 Product Complaint

11.2.1 Definition

A Product Complaint is any Complaint related to the biologic or drug component of the product or to the medical device component(s).

For a product this may include, but is not limited to, damaged/broken product or packaging, product appearance whose color/markings do not match the labeling, labeling discrepancies/inadequacies in the labeling/instructions (example: printing illegible), missing components/product, device not working properly, or packaging issues.

For medical devices, a product complaint also includes all deaths of a patient using the device, any illness, injury, or adverse event in the proximity of the device, an adverse event that could be a result of using the device, any event needing medical or surgical intervention including hospitalization while using the device and use errors.

Any information available to help in the determination of causality by the device to the events outlined directly above should be reported.

11.2.2 Complaint Reporting

Product Complaints concerning an AbbVie authorized product and/or device must be reported to AbbVie. The AbbVie contact details are specified in Country Information page (Section 12.0).

Product complaints involving a non-AbbVie product and/or device should be reported to the identified contact or manufacturer, as necessary per local regulations.

Product Complaints may require return of the product with the alleged complaint condition (syringe, pen, etc.) as per standard process.

The description of the complaint is important for AbbVie in order to enable AbbVie to investigate and determine if any corrective actions are required.

12.0 Country Information Page

AbbVie
Cross-sectional Study Evaluating patieNt satisfaction, adherence featureS, and thEir association with socio-demographic and clinical characteristics of DMARD-inadequate responder rheumatoid arthritis patients – SENSE

Protocol H17-155

Sponsor:

Name Medical Director:
Address:
Address:
Country:
Phone:
Fax:

Safety/Pregnancy Reporting to:

E-mail:
Phone:
Fax:

Complaints Reporting to:

E-mail:
Phone:
Fax:

Shipment address for questionnaires:

Name:
Address:
Address:
Country:
Phone:
Fax:

Requirements for Non-Interventional Studies per Local Laws and Regulations:

Competent Authority approval ☐

Competent Authority notification ☐

Competent Authority involvement not required ☐

Ethics Committee approval ☐

Ethics Committee notification ☐

Ethics Committee involvement not required ☐

Written Patient Informed Consent required: ☐ No* ☐ Yes

*Written Patient Authorization to Disclose Data still required.

Regulatory requirements, other (if applicable):

____

____

Name of Affiliate Medical Director or equivalent role (i.e., Regulatory for US)

____ ______________

Signature Date

13.0 Plans for Disseminating and Communicating Study Results

At the end of the study, a report or publication will be written by AbbVie. This report or publication will contain a description of the objectives of the study, the methodology and its results and conclusions. The completed eCRFs, questionnaires and the final study output are the confidential property of AbbVie and may not be released to unauthorized people in any form (publications or presentations) without the express written approval from AbbVie.

14.0 References

1. Lee DM, Weinblatt ME. Rheumatoid arthritis. Lancet, 2001; 358:903-911.
2. Scott DL, Wolfe F, Huizinga TWJ. Rheumatoid arthritis. Lancet, 2010; 376:1094-1108.
3. Elliott RA. Poor adherence to medications in adults with rheumatoid arthritis. Dis Management Health Outcomes 2008; 16(1):13-29.
4. S. Joplin S, van der Zwan R, Joshua F, Wong PKK. Medication adherence in patients with rheumatoid arthritis: The effect of patient education, health literacy, and musculoskeletal ultrasound. BioMed Res International, 2015; Doi:10.1155/2015/150658.
5. Rubbert-Roth A, Finckh A. Treatment options in patients with rheumatoid arthritis failing initial TNF inhibitor therapy: a critical review. Arthritis Res Ther 2009; 11(Suppl 1):S1.
6. Nagy G, van Vollenhoven RF. Sustained biologic-free and drug-free remission in rheumatoid arthritis, where are we now? Arthritis Res Ther 2015; 17:181.
7. van der Helm-van Mil AH, Knevel R, Cavet G, et al. An evaluation of molecular and clinical remission in rheumatoid arthritis by assessing radiographic progression. Rheumatology (Oxford) 2013; 52:839–46.
8. Smolen JS, Landewe R, Breedveld FC, et al. EULAR recommendations for the management of rheumatoid arthritis with synthetic and biological disease-modifying antirheumatic drugs: 2013 update. Ann Rheum Dis 2014;73:492–509.
9. Radner H, Smolen JS, Aletaha D. Remission in rheumatoid arthritis: benefit over low disease activity in patient-reported outcomes and costs. Arthritis Res Ther 2014;16:R56.
10. Louder AM, Singh A, Saverno K, et al. Patient Preferences regarding rheumatoid arthritis therapies: A conjoint analysis. Am Health Drug Benefits 2016;9:84–93.

11. Buch MH, Marzo-Ortega H, Bingham SJ, et al. Long-term treatment of rheumatoid arthritis with tumour necrosis factor alpha blockade: outcome of ceasing and restarting biologicals. Rheumatology (Oxford) 2004;43:243–244.

12. Jacobs JW. Lessons for the use of non-biologic anchor treatments for rheumatoid arthritis in the era of biologic therapies. Rheumatology (Oxford). 2012;51(Suppl 4):iv27–33.

13. Wollenhaupt J, Silverfield J, Lee EB, et al. Tofacitinib, an oral Janus kinase inhibitor, for the treatment of rheumatoid arthritis: safety and efficacy in open-label, long-term extension up to 6 years. Ann Rheum Dis 2015;74(Suppl 2):259.

14. Genovese MC, Kremer J, Zamani O, et al. Baricitinib in Patients with Refractory Rheumatoid Arthritis N Engl J Med. 2016 Mar 31;374(13):1243-52. doi: 10.1056/NEJMoa1507247.

15. Keystone EC, Taylor PC, Drescher E, et al. Safety and efficacy of baricitinib at 24 weeks in patients with rheumatoid arthritis who have had an inadequate response to methotrexate. Ann Rheum Dis 2015;74:333–340.

16. Dougados M, van der Heijde D, Chen Y-C, et al. Baricitinib, an oral janus kinase (JAK)1/JAK2 inhibitor, in patients with active rheumatoid arthritis (RA) and an inadequate response to cDMARD therapy: results of the Phase 3 RA-BUILD study. Ann Rheum Dis 2015;74(Suppl 2):79 [Abstract LB0001].

17. Fleischmann R, Kremer J, Cush J, et al. Placebo-controlled trial of tofacitinib monotherapy in rheumatoid arthritis. N Engl J Med 2012;367:495–507.

18. van Vollenhoven RF, Fleischmann R, Cohen S, et al. Tofacitinib or adalimumab versus placebo in rheumatoid arthritis. N Engl J Med 2012;367:508–519.

19. Burmester GR, Blanco R, Charles-Schoeman C, et al. Tofacitinib (CP-690,550) in combination with methotrexate in patients with active rheumatoid arthritis with an inadequate response to tumour necrosis factor inhibitors: a randomised phase 3 trial. Lancet 2013;381:451–460.

20. van der Heijde D, Tanaka Y, Fleischmann R, et al. Tofacitinib (CP-690,550) in patients with rheumatoid arthritis receiving methotrexate: twelve-month data from a twenty-four-month phase III randomized radiographic study. Arthritis Rheum 2013;65:559–570.

21. Tsuchida T. Is tofacitinib effective for elderly patients (aged 70 years and older) with rheumatoid arthritis? Ann Rheum Dis 2015;74(Suppl 2):1070 [Abstract AB0512].

22. Fleischmann R, Takeuchi T, Schlichting DE, et al. Baricitinib, methotrexate, or baricitinib plus methotrexate in patients with early rheumatoid arthritis who had received limited or no treatment with

disease-modifying anti-rheumatic drugs (DMARDs): Phase 3 trial results [abstract]. Arthritis Rheumatol 2015;67 (Suppl 10).

23. Smolen JS, Landewé R, Bijlsma J, et al. EULAR recommendations for the management of rheumatoid arthritis with synthetic and biological disease-modifying antirheumatic drugs: 2016 update. Ann Rheum Dis, 2017 Jun;76(6):960-977.

24. Kremer JM, Keystone EC, Emery P, et al. Safety and efficacy of ABT-494, a novel selective JAK1 Inhibitor, in patients with active rheumatoid arthritis and inadequate response or intolerance to anti-TNF biologic therapy. Arthritis Rheumatol 2015;67(Suppl 10):3962–3963 [Abstract 14L].

25. Genovese M, Smolen J, Weinblatt M, et al. A randomized phase 2b study of ABT-494, a selective JAK1 inhibitor in patients with rheumatoid arthritis and an inadequate response to methotrexate. Arthritis Rheumatol 2016;68:2857-2866.

26. Kremer J, Emery P, Camp H, et al. A phase 2b study of ABT-494, a selective JAK1 inhibitor, in patients with rheumatoid arthritis and an inadequate response to anti–TNF therapy. Arthritis Rheumatol. 2016;68:2867-2877.

27. Thier SL et al. In chronic disease, nationwide data show poor adherence by patients to medication and by physicians to guidelines. Manag Care 2008;17(2):48-52, 55-57.

28. Sabaté E. Adherence in long-term therapy; evidence for action. World Health Organization 2003. Source: http://apps.who.int/iris/bitstream/10665/42682/1/9241545992.pdf, Accessed: 29 November 2017.

29. Harrold LR et al. Medication adherence of patients with selected rheumatic conditions: a systematic review of the literature. Semin Arthritis Rheum 2009;38(5):396-40.

30. Van den Bosch F et al. Impact of Patient Support Program Use on Clinical Outcomes Among Patients With Rheumatoid Arthritis. Ann Rheum Dis 2015;74(Suppl 2):227. Poster presented at: 16th Annual European League Against Rheumatism (EULAR); June 10-13, 2015; Rome, Italy. Abstract THU0097.

31. Rubin DT et al. Impact of AbbVie's Patient Support Program on Resource Costs in Crohn's Disease, Ulcerati ve Colitis, Rheumatoid Arthritis, Psoriasis, Psoriatic Arthritis, and Ankylosing Spondylitis. Poster presented at the Academy of Managed Care Pharmacy (AMCP), San Diego, California, April 7-10, 2015.Presented at the Academy of Managed Care Pharmacy (AMCP), San Diego, California, April 7-10, 2015.

32. Koncz T et al. Adherence to biologic DMARD therapies in rheumatoid arthritis. Expert Opin. Biol. Ther. 2010;10(9):1367-1378

33. van den Bemt BJF et al. Medication adherence in patients with rheumatoid arthritis: a critical appraisal of the existing literature. Expert Rev Clin Immunol, 2012;8(4):337-351.

34. Lam WY, Fresco P. Medication Adherence Measures: An Overview. BioMed Res Intenational, 2015; 2015;2015:217047. doi: 10.1155/2015/217047

35. Hughes LD, Done J, Young A.A 5 item version of the Compliance Questionnaire for Rheumatology (CQR5) successfully identifies low adherence to DMARDs. BMC Musculosceletal Disorders, 2013; 14:286.

36. Grijalva CG, Chung CP, Arbogast PG, Stein CM, Mitchel EF, Griffin MR. Assessment of adherence to and persistence on Disease-Modifying Antirheumatic Drugs (DMARDs) in patients with rheumatoid arthritis. Med Care, 2007; 45(10):566.

37. Singh JA, et al. 2015 American College of Rheumatology Guideline for the Treatment of Rheuamtoid arthritis. Arthritis Rheumatol. 2016;68(1):1-26.

38. Yazici Y. Abatacept versus other biologics in methotrexate inadequate responders with rheumatoid arthritis: you like tomato and I like tomatho…let's call the whole off. Arthritis Res Ther. 2012;14(1):104.

39. Alten R. et al. Examining patient preferences in the treatment of rheumatoid arthritis using a discrete-choice approach. Patient Preference and Adherence, 2016:10 2217-2228.

40. Michetti,P et al. Impact of treatment-related beliefs on medication adherence in immune-mediated inflammatory diseases: results of the global ALIGN study. Adv Ther, 2017; 34:91-108.

41. Norman CD, Skinner HA. eHEALs:The eHEALs literacy scale. J Med Internet Res. 2006 Oct-Dec; 8(4): e27.

42. Ganguli A et al. The impact of patient support programs on adherence, clinical, humanistic, and economic patient outcomes: a targeted systematic review. Patient Prefer Adher. 2016;10:711–25.

43. Stockl KM, Shin JS, Lew HC, et al. Outcomes of a rheumatoid arthritis disease therapy management program focusing on medication adherence. J Manag Care Pharm. 2010;16:593–604.

44. Jackson JL, Chamberlin J, Kroenke K. Predictors of patient satisfaction. Soc Sci Med 2001;52:609–20.

45. McCracken LM, Evon D, Karapas ET. Satisfaction with treatment for chronic pain in a specialty service: preliminary prospective results. Eur J Pain 2002;6:387–93.

46. Shikiar R, Halpern MT, McGann M, et al. The relation of patient satisfaction with treatment of otitis externa to clinical outcomes: development of an instrument. Clin Ther 1999;21:1091–104.

47. Shikiar R, Rentz AM. Satisfaction with medication: an overview of conceptual, methodologic, and regulatory issues. Value Health 2004;7:204–15.

48. van Den Bosch F, Wassenberg S, Östör A, et al. Impact of participation in adalimumab patient support program (PSP) on rheumatoid arthritis treatment course: results from the PASSION study. Rheumatol Ther 2017;4:85-96.

49. Levy RA, et al. Patient Support Program for Adalimumab-Treated Patients in Brazil: Impact on Patients' Adherence and Persistence. Arthritis Rheumatol 2016; 68 (Suppl. 10):Abstract 100.

50. Srulovici E, et al. Is Patient Support Program (PSP) Participation Associated with Longer Persistence and Greater Adherence Among New Users of Adalimumab? Arthritis Rheumatol 2016; 68 (Suppl. 10):Abstract 92.

51. Mark Atkinson et al. Validation of a general measure of treatment satisfaction, the Treatment Satisfaction Questionnaire for Medication (TSQM), using a national panel study of chronic disease. Health and Quality of Life Outcomes 2004, 2:12.

52. Aletaha D, Smolen JS. What should be our treatment goal in rheumatoid arthritis today? Clin Exp Rheum 2006;24(Suppl. 43):S45–S51.

53. Cunha-Mirande et al, NEAR study: Needs and Expectations in rheumatoid ARthritis- do we know our patient's needs? Acta Rheumatol Port 2010;35:314-323.

54. Smolen JS, Breedveld FC, Burmester GR, et al Treating rheumatoid arthritis to target: 2014 update of the recommendations of an international task force Annals of the Rheumatic Diseases Published Online First: 12 May 2015. doi: 10.1136/annrheumdis-2015-207524

55. Meenan RF, Gertman PM, Mason JH. Measuring health status in arthritis. The arthritis impact measurement scales. Arthritis Rheum. 1980 Feb;23(2):146-52.

56. Orbai AM, Bingham CO III. Patient reported outcomes in rheumatoid arthritis clinical trials. Curr Rheumatol Rep 2015;17:28

57. Gossec L, Dougados M, Dixon W. Patient-reported outcomes as end points in clinical trials in rheumatoid arthritis. RMD Open 2015;1(1):e000019.

58. Fries JF, Spitz P, Kraines RG et al. Measurement of patient outcome in arthritis. Arthritis Rheum1980;23:137–45.

59. Sokka T. Morning stiffness and other patient-reported outcomes of rheumatoid arthritis in clinical practice. Scand J Rheumatol Suppl 2011;125:23–7.

60. Avlund K. et al. Functional decline from age 80 to 85: Influence of preceding changes in tiredness in daily activities. Psychosomatic Medicine, 2003;65, 771-777.

61. Carr A, Hewlett S, Hughes R, et al. Rheumatology outcomes: the patient's perspective. J Rheumatol 2003;30:880–3.

62. Hewlett S, Cockshott Z, Byron M, et al. Patients’ perceptions of fatigue in rheumatoid arthritis: overwhelming, uncontrollable, ignored. Arthritis Rheum 2005;53:697–702.

63. SokkaT. Morning stiffness and other patient-reported outcomes of rheumatoid arthritis in clinical practice. Scand J Rheumatol Suppl. 2011;125:23-7.

64. YaziciY et al. Morning stiffness in patients with early rheumatoid arthritis is associated more strongly with functional disability than with joint swelling and erythrocyte sedimentation rate. J Rheumatol. 2004;31(9):1723-6.

65. Walsh DA, McWilliams DF. Mechanisms, impact and management of pain in rheumatoid arthritis. Nat Rev Rheumatol. 2014 Oct;10(10):581-92.

66. van der Heijde DMFM et al.: Judging disease activity in clinical practice in rheumatoid arthritis: first step in the development of a disease activity score. Ann Rheum Dis 1990; 49: 916-20.

67. Van der Heijde et al. Development of a disease activity score based on judgment in clinical practice by rheumatologists. J Rheumatol 1993;20:579-581.

68. Kushner I. C-reactive protein in rheumatology. Arthritis Rheum 1991;34:1065–8.

69. Smolen J. et al. A simplified disease activity index for rheumatoid arthritis for use in clinical practice. Rheumatology (Oxford) 2003;42:244- 57.

70. Aletaha D. et al. Acute phase reactants add little to composite disease activity indices for rheumatoid arthritis: validation of a clinical activity score. Arthritis Res Ther 2005; 7: R796-R806.

71. Buchbinder R et al. Which outcome measures should be used in rheumatoid arthritis clinical trials? Clinical and quality-of-life measures' responsiveness to treatment in a randomized controlled trial. Arthritis Rheum 1995; 38:1568–80.

72. Webster K. et al. The functional assessment of chronic illness therapy (FACIT) measurement system: Properties, applications, and interpretation. Health and Quality of Life Outcomes, 2003; 1(79), 1-7.

73. http://www.quintiles.com/landing-pages/treatment-satisfaction-questionnaire-for-medication-tsqm Accessed at 29 November 2017.

74. Walsh JC et al. Responses to a 1 month self-report on adherence to antiretroviral therapy are consistent with electronic data and virological treatment outcome. AIDS. 2002 Jan 25;16(2):269-77.

75. Prasad M et al. A review of self-report instruments measuring health-related work productivity: a patient-reported outcomes perspective. Pharmacoeconomics. 2004;22(4):225-44.

76. Source: http://www.qualitymetric.com/WhatWeDo/SFHealthSurveys/SF36v2HealthSurvey/tabid/185/Default.aspx Accessed: 29 November 2017.

77. Mathijssen E et al. Rheumatoid arthritis patients support needs regarding medication use and their perspectives on the applicability of e-helath interventions to address those needs: a focus group study. Abstract presented at EULAR 2017 congress, OP0145. 10.1136/annrheumdis-2017-eular.6344

78 Taylor PC et al. A structured literature review of the burden of illness and unmet needs in patients with rheumatoid arthritis: a current perspective. Rheumatol Int .2016; 36:685–695.

79. Zuidema RM et al. An Online Tailored Self-Management Program for Patients With Rheumatoid Arthritis: A Developmental Study.JMIR Res Protoc. 2015 Dec 25;4(4):e140.

80 Kobelt G.et al. Costs and quality of life of patients with ankylosing spondylitis in Canada. J Rheumatol 2006; 33: 289-95.

81. Mersdal S, et al. Indirect cost assessment in patients with rheumatoid arthritis (RA): comparison of data from the health economic patient questionnaire HEQ-RA and insurance claims data. Arthritis Rheum 2005; 53: 234-40.

82. Severens JL, et al. Precision and accuracy in measuring absence from work as a basis for calculating productivity costs in The Netherlands. Soc Sci Med 2000; 51: 243-9.

83. Paresh Jobanputra et al. A randomised efficacy and discontinuation study of etanercept versus adalimumab (RED SEA) for rheumatoid arthritis: a pragmatic, unblinded, non-inferiority study of first TNF inhibitor use: outcomes over 2 years. BMJ Open 2012;2:e001395

84. Michetti P. Et al. Muli-Country, Cross-Sectional Study to Determine Patent-Specific and General Beliefs Toward Medicaon and Their Treatment Adherence to Selected Systemic Therapies in 6 Chronic Immune-Mediated Inflammatory Diseases (ALIGN). Poster presented at the United European Gastroenterology Week 22nd Annual Conference, 18-22 October 2014, Vienna, Austria. Abstract#P1430.

15.0 Appendices

Appendix 1: List of copyright protected patient reported outcome questionnaires

- Disease activity measures: DAS28, CDAI, SDAI
- Morning stiffness and joint pain measures
- HAQ-DI
- Functional Assessment of Chronic Illness Therapy (FACIT) Fatigue Scale (Version 4)
- TSQM version 1.4
- WPAI-RA
- SF-36v2
- eHEALS

Appendix 2: Self – reported adherence

Put a mark at the line below showing your best guess about how much of your medication you have taken within the last 3 months.

0% means you have taken no medicine

50% means you have taken half of the prescribed doses

100% means you have taken all prescribed doses

0% 50% 100%

Appendix 3: Questionnaire to assess patient needs for support via PSP

This survey asks your needs related a patient support program* (PSP) for your RA. Please see the short description of PSP in the end of the survey.

For each questions, please cselect one ansver which reflect your needs most likely.
Please use a scale of 1 to 7 where 1 is 'Not needed at all', 4 is 'Unsure' and 7 is 'Very much needed'

	1 ***Not needed at all***	**2**	**3**	***4*** ***Unsure***	**5**	**6**	***7*** ***Very much needed***
**Do you need a Patient Support Program in general?**	1	2	3	4	5	6	7
**Do you need a Call center/Hotline?**	1	2	3	4	5	6	7
**Do you need Educational materials about RA disease?**	1	2	3	4	5	6	7
**Do you need Educational materials about the therapy you take for RA?**	1	2	3	4	5	6	7
**Do you need Nursing service?**	1	2	3	4	5	6	7
**Do you need personalized Care coach?**	1	2	3	4	5	6	7
**Do you need a Starter pack with all information about the patient support program?**	1	2	3	4	5	6	7
**Do you need an e-mail contact during the patient support program?**	1	2	3	4	5	6	7
**Do you need medication administration reminders?**	1	2	3	4	5	6	7
**Do you need Doctor Appointment Reminders?**	1	2	3	4	5	6	7
**Do you need educational materials about everyday coping with the disease?**	1	2	3	4	5	6	7
**Do you need information about everyday coping with the disease?**	1	2	3	4	5	6	7

**Do you need digital lifestyle intervention?**	1	2	3	4	5	6	7
**Do you need mental or emotional support?**	1	2	3	4	5	6	7
**Do you need a website where all information available related to the patient support program?**	1	2	3	4	5	6	7
**Do you need a social media communication channel related to the patient support program?**	1	2	3	4	5	6	7
**Do you need a smartphone application related to the patient support program?**	1	2	3	4	5	6	7

* Patient support programs (PSPs) are enhanced self-management support programs that include interventions such as individualized medication counseling, training, support, and virtual reminders to improve medication-taking behavior. The underlying objective is to help patients better manage their disease and complex medication regimens, improve medication adherence, and reduce complications and related costs.

Appendix 4: Questionnaire to assess medication preferences

We would like to ask you about your preferences regarding medication used for rheumatoid arthritis. For each questions, please circle one ansver which reflect your opinion most likely.

1. What is the referred route of administration?
    a. Parenteral: Intravenous
    b. Parenteral: Subcutaneous
    c. Oral
2. What is the preferred frequency of administration in case of parenteral administration?
    a. Biweekly
    b. Monthly
    c. 3-monthly
    d. 6-monthly
3. What is the preferred frequency of administration in case of oral administration?
    a. Twice per day
    b. Once per day
    c. Once per week
4. What is the preferred time until the effect of onset?
    a. Up to 1 week
    b. Up to 2 weeks
    c. Up to 1 months
    d. Up to 3 months
5. What is your preference regarding drug combinations used for your rheumatoid arthritis?
    a. Drug combination is not preferred.
    b. Treatment which requires daily combination is acceptable.
    c. Treatment which requires combination with another drug once a week is acceptable.
6. What is the most acceptable potential side effect of the medication used for rheumatoid arthritis?
    a. Increased risks for infections
    b. Allergic reaction
    c. Deterioration of my laboratory values
    d. Increased risk for malignancies
    e. Weight gain
    f. Hair thinning or loss
    g. Skin symptoms e.g. injection site reaction, rash
    h. Effect on fertility
    i. Increased risk of cardiovascular diseases

Appendix 5: Questionnaire to assess treatment expectations

Please take some time to think about the key areas of your health related to RA in which you would like to see improvement.
Please use a scale of 1 to 7 where 1 is 'No improvement needed', 4 is 'Some improvement needed', and 7 is 'The most improvement needed'

	1 *No improvement needed*	**2**	**3**	***4*** *Some improvement needed*	**5**	**6**	***7*** *The most improvement needed*
**General improvement of arthritis**	1	2	3	4	5	6	7
**Less joint pain**	1	2	3	4	5	6	7
**Less joint swelling**	1	2	3	4	5	6	7
**Lasting relief of RA symptoms**	1	2	3	4	5	6	7
**More joint flexibility**	1	2	3	4	5	6	7
**Improvement in morning stiffness in the limbs**	1	2	3	4	5	6	7
**Less tiredness and less fatigue**	1	2	3	4	5	6	7
**Improvement in mood**	1	2	3	4	5	6	7
**Improvement in self-care**	1	2	3	4	5	6	7
**Improvement in workability**	1	2	3	4	5	6	7
**Improvements in sleep**	1	2	3	4	5	6	7

Appendix 6: Health care resource utilization (HRU) in the past 3 months from enrollment

In the past 3 months, has the subject had any medical visits for their rheumatoid arthritis (RA) ?

YES: ____
NO: ____
If YES, please provide the following:

1. In the past 3 months, has the subject been seen by a health care professional for their RA?

YES: ____
NO: ____
If YES, how many times: ____

2. In the past 3 months, has the subject been seen in the Emergency Room for their RA?

YES: ____
NO: ____
If YES, how many times: ____

3. In the past 3 months, has the subject been admitted to the hospital due to their RA?

YES: ____
NO: ____
If YES, please list the ADMISSION DATE: __ __/___ ___ ___/ ___ ___ ___ __
DD MMM YYYY
DISCHARGE DATE: __ __/___ ___ ___/ ___ ___ ___ __
DD MMM YYYY

Appendix 7: List of changes in Amendment #01

The purpose of this Amendment is to:

- Revise study milestones
- Revise sample size calculation based on country projections on enrollment
- Removal of copyright protected PRO questionnaires from the Appendix
- Correct typos

Section Number and Title: 5.0 Milestones
Previously read:

Major study milestones and their planned dates are as follows:

Start of Data Collection:	02 August 2018
End of Data Collection:	01 May 2019
Final Report of Study Results:	01 December 2019

Has been changed to read:

Major study milestones and their planned dates are as follows:

Start of Data Collection:	02 September 2018
End of Data Collection:	31 May 2019
Final Report of Study Results:	01 December 2019

Section Number and Title: Study size 9.3
Previously read:

Approximately 2000 RA patients will be included in the study. The study will include 50 sites in approximately 20 countries in Europe, America and Asia. Each country will recruit approximately 100 patients.

Current sample size calculation is based on information of global satisfaction measured by TSQM 1.4. It is also assumed that similar number of patients will be allocated to each country so that country level analyses will be explored, as well as overall population. Based on the literature [51, 83], it seems reasonable to assume that the standard deviation for the global satisfaction is about 20 with mean value range from 71 – 91.

A given sample size of N=100 at each country, will be able to provide a 95% confidence interval (CI) with a half width of 4.0 At this level, 20 countries will enroll N=2000 patients in total, this will allow half width of 95% CI to be 0.88 in the overall study population.

Has been changed to read:

Approximately 1500 RA patients will be included in the study. The study will include 100 sites in approximately 20 countries in Europe, America and Asia. Countries will recruit between 30 and 200 patients per country depending on the available patient population.

Current sample size calculation is based on information of global satisfaction measured by TSQM 1.4. It is also assumed that similar number of patients will be allocated to each country so that country level analyses will be explored, as well as overall population. Based on the literature [51, 83], it seems reasonable to assume that the standard deviation for the global satisfaction is about 20 with mean value range from 71 – 91.

A given sample size of N=1500 will be able to provide a 95% confidence interval (CI) with a half width of 1.01 in the overall study population.

For country specific analysis a sample of N=50 will be able to provide a 95% confidence interval (CI) with a half width of 5.68. For country specific analysis a sample of N=30 – 200 will be able to provide a 95% confidence interval (CI) with a half width of 7.47 to 2.79.

AbbVie

Non-Interventional Study (Non-PMOS)

Cross-sectional Study Evaluating patieNt satisfaction, adherence featureS, and thEir association with socio-demographic and clinical characteristics of DMARD-inadequate responder rheumatoid arthritis patients- SENSE

Approved by:

Protocol Author - Orsolya Nagy, EEMEA Rheumatology Medical Lead

21 March 2019
Date

TA MD/SD or equivalent - John Farrell, EEMEA Medical Head

27nd March 2019
Date
